# HMGB1-mediated formation of IL-33–abundant NETs drives lung-to-kidney injury in severe pneumonia–associated acute kidney injury

**DOI:** 10.1172/jci.insight.191979

**Published:** 2026-05-22

**Authors:** Mengqing Ma, Hao Zhang, Weijuan Deng, Xia Du, Mengxing Chen, Dawei Chen, Binbin Pan, Zhaowei Wang, Ting Chen, Caimei Chen, Xin Wan, Changchun Cao

**Affiliations:** 1 Department of Nephrology, Nanjing First Hospital, Nanjing Medical University, Nanjing, Jiangsu, China.; ² Department of Nephrology, Nanjing First Hospital, Nanjing University of Chinese Medicine, Nanjing, Jiangsu, China.; ³ Department of Nephrology, Sir Run Run Hospital, Nanjing Medical University, Nanjing, Jiangsu, China.; 4 Department of Nephrology, Jiujiang No. 1 People’s Hospital, Jiujiang, Jiangxi, China.; 5 Department of Nephrology, Wuxi People’s Hospital Affiliated with Nanjing Medical University, Wuxi, Jiangsu, China.

**Keywords:** Inflammation, Nephrology, Pulmonology, Cellular immune response, Neutrophils, Urology

## Abstract

Acute kidney injury (AKI) is a common and fatal complication of severe pneumonia, yet the mechanisms linking pulmonary inflammation to remote kidney injury remain poorly understood. Multicenter cohort data (*n* = 300) revealed that the incidence of severe pneumonia–associated AKI (SP-AKI) was 53.6%, with a mortality rate of 24.2%. SP-AKI was associated with elevated circulating levels of HMGB1, NETs, and IL-33. Murine experiments demonstrated that alveolar HMGB1 triggers the formation of IL-33–enriched NETs, which migrate to the kidney and activate tubular ST2/NF-κB signaling, driving inflammation and apoptosis. Genetic knockout of IL-33, ST2, or the NET-forming key enzyme PAD4, as well as pharmacological inhibition of HMGB1, IL-33, or NETs, all attenuated lung and kidney injury. Exogenous HMGB1 amplified NET-mediated IL-33 release, establishing a self-sustaining HMGB1/NET/IL-33 feed-forward loop. PAD4 deficiency completely blocked NET generation and disrupted HMGB1/IL-33 signaling. This study identified and validated a damage-associated molecular pattern–driven (DAMP-driven) HMGB1/NET/IL-33 signaling axis that mediates remote kidney injury in SP-AKI, redefining NETs from local effectors to cross-organ pathogenic carriers, thereby providing potential DAMP-targeted therapeutic avenues for SP-AKI.

## Abstract 2

Lung-derived HMGB1 induces the formation of IL-33–enriched neutrophil extracellular traps (NETs), which circulate to activate renal ST2/NF-κB signaling and cause tubular injury.

## Graphical abstract

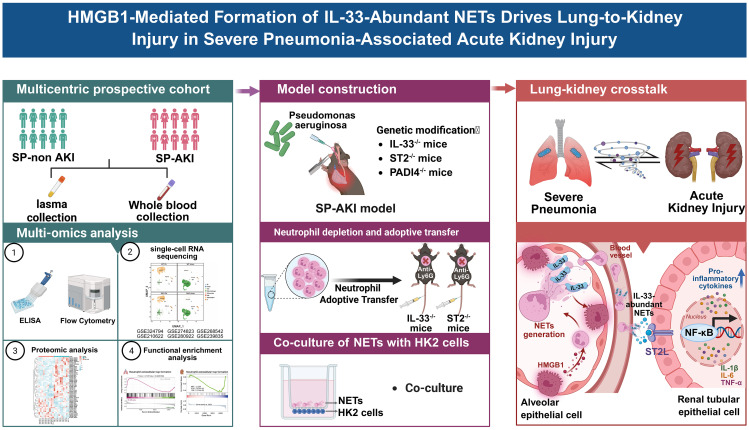

## Introduction

Acute kidney injury (AKI) is a frequent and life-threatening complication in patients with severe pneumonia, substantially increasing short-term mortality and adversely affecting long-term outcomes (1, 2). Despite advances in antimicrobial therapy and organ support, the incidence of severe pneumonia–associated AKI (SP-AKI) remains unacceptably high, and effective disease-modifying treatments are still lacking. This sobering clinical reality highlights a fundamental unresolved question: How does lung injury transmit inflammatory signals to the kidney and drive remote organ damage?

Traditionally, renal injury in severe pneumonia has been attributed primarily to systemic hemodynamic instability, hypoxia, and infection-related pathogen-associated molecular patterns (PAMPs) (3). However, accumulating clinical and experimental evidence indicates that damage-associated molecular patterns (DAMPs) play a central role in mediating remote organ injury (4). In this context, the lung-kidney axis has emerged as a critical determinant of outcomes in critically ill patients, yet the key molecular mediators that convey inflammatory signals from injured lungs to the kidney remain incompletely defined.

In recent years, neutrophils and neutrophil extracellular traps (NETs) have been widely recognized as major amplifiers of tissue injury in acute inflammatory diseases (5, 6). Beyond their antimicrobial function, excessive NET formation contributes to endothelial dysfunction, microvascular damage, and epithelial toxicity, exerting pathogenic effects in both acute lung injury and AKI (7, 8). Nevertheless, most previous studies have focused on the local actions of NETs, and whether NETs generated in injured lungs can function as systemic mediators that propagate renal injury remains largely unexplored.

Among the diverse repertoire of DAMPs, high-mobility group box 1 (HMGB1) has been identified as a key regulator of inflammation. HMGB1 is passively released during cell necrosis and orchestrates leukocyte recruitment, cytokine release, and NET formation. A substantial body of evidence demonstrates that HMGB1 drives NET generation through pattern recognition receptors such as TLR4, and that pharmacological inhibition of HMGB1 effectively attenuates inflammatory tissue damage (9). In parallel, the alarmin cytokine IL-33 has gained increasing attention for its role in acute lung injury, sepsis, and kidney disease (10–12). Notably, recent studies suggest that IL-33 can be processed and released in the context of NET formation, raising a compelling possibility: NETs may serve not only as inflammatory effector structures but also as carriers of bioactive cytokines (13).

In the present study, we integrated a multicenter clinical cohort with multiomics analyses and complementary genetic and pharmacological approaches in murine models to systematically investigate lung-kidney inflammatory crosstalk in severe pneumonia. We identified and validated a DAMP-driven HMGB1/NET/IL-33 signaling axis and demonstrated its pivotal role in mediating remote renal injury in SP-AKI.

## Results

### Proteomics measurement and bioinformatics analysis conducted in patients with severe pneumonia.

**Figure 1:**
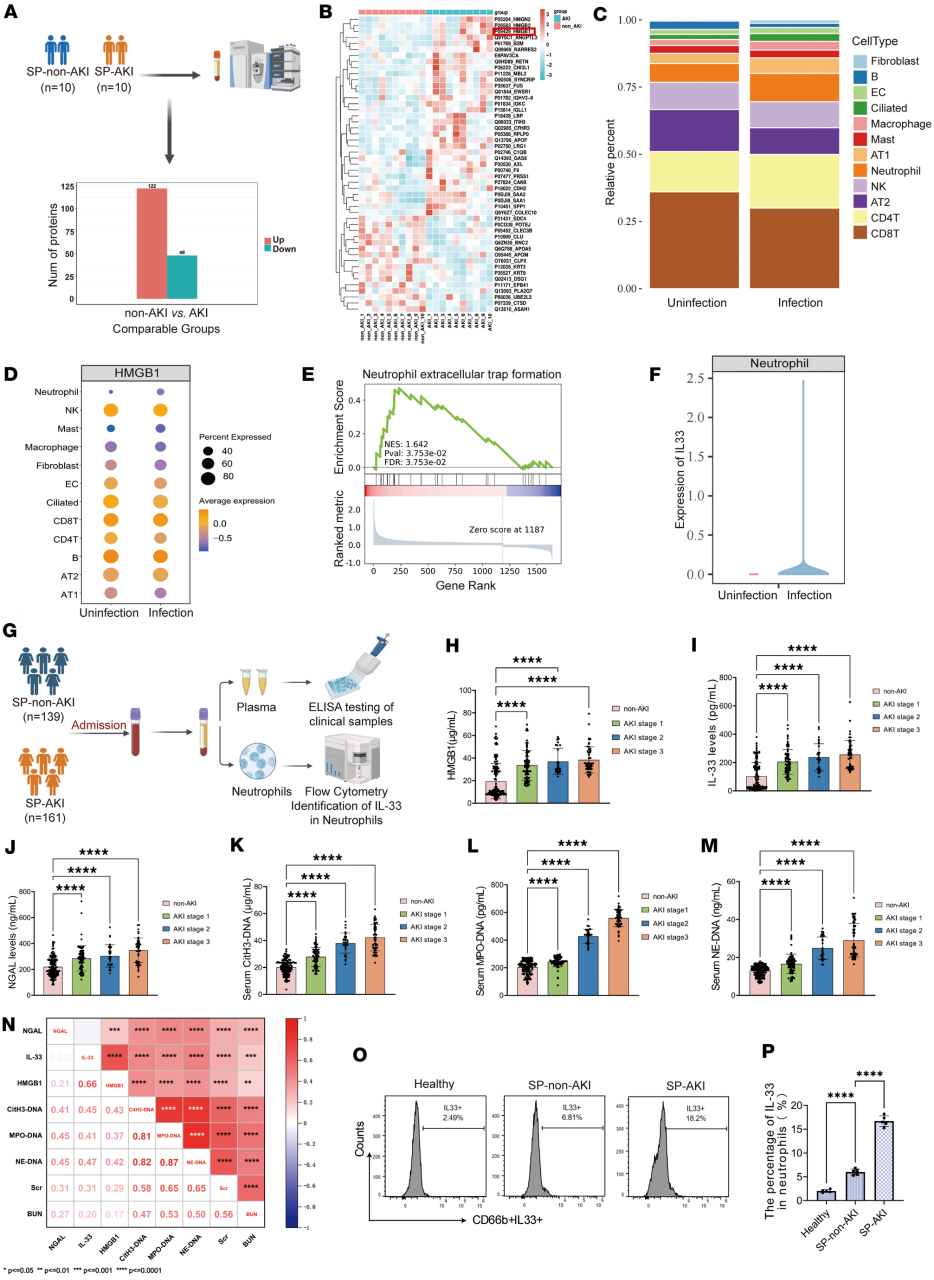
Elevated serum HMGB1, NET, and IL-33 levels are associated with SP-AKI in patients. (**A**) Schematic of serum proteomic analysis comparing patients with SP–non-AKI (*n* = 10) and SP-AKI (*n* = 10), showing upregulated and downregulated serum proteins. (**B**) Heatmap of differentially expressed proteins between SP–non-AKI and SP-AKI, with hierarchical clustering. Red, upregulated; blue, downregulated. (**C**) Public single-cell transcriptomic analysis of human lungs with bacterial and viral infections reveals major pulmonary cell populations (GSE268542). (**D**) Dot plot showing high-mobility group box 1 (HMGB1) expression across lung cell types in uninfected versus infected conditions. (**E**) Gene set enrichment analysis demonstrating significant enrichment of the neutrophil extracellular trap (NET) formation pathway. Normalized enrichment score (NES) and false discovery rate (FDR) are shown. (**F**) Violin plot showing IL-33 expression in neutrophils under uninfected and infected conditions. (**G**) Schematic diagram. Blood samples were collected upon admission from patients with SP-non-AKI (*n* = 139) and SP-AKI (*n* = 161), including ELISA measurement of serum HMGB1, IL-33, neutrophil gelatinase-associated lipocalin (NGAL), and NET-DNA complex levels, as well as flow cytometric analysis. (**H**) Serum concentrations of HMGB1 in patients without AKI and in patients with AKI stages 1–3 (total *n* = 300). (**I**–**M**) Serum levels of IL-33 (**I**), NGAL (**J**), citrullinated histone H3–DNA (CitH3-DNA) complexes (**K**), myeloperoxidase-DNA (MPO-DNA) complexes (**L**), and neutrophil elastase–DNA (NE-DNA) complexes (**M**). (**N**) Correlation matrix showing associations among circulating biomarkers, including IL-33, HMGB1, NGAL, NET-DNA markers, and clinical indices. (**O** and **P**) Representative flow cytometry plots and quantitative analysis of IL-33^+^ neutrophils (CD66b^+^IL-33^+^) in peripheral blood from healthy controls, SP–non-AKI patients, and SP-AKI patients (*n* = 5). Data are presented as mean ± SD. *P* values were calculated by 1-way ANOVA with post hoc Holm-Šídák test. ****P < 0.0001.

To identify molecular pathways associated with SP-AKI, we performed serum proteomic profiling in 10 patients with SP-AKI and 10 matched non-AKI controls. A total of 170 differentially expressed proteins were identified (122 upregulated, 48 downregulated) (Figure 1A). Notably, HMGB1 and HMGB2 ranked among the top 10 upregulated proteins, suggesting their potential involvement in SP-AKI pathogenesis (Figure 1B). Gene Ontology enrichment analysis revealed significant enrichment of immune-related biological processes, extracellular compartment localization, and binding-related molecular functions (Supplemental Figure 1A; supplemental material available online with this article; https://doi.org/10.1172/jci.insight.191979DS1). Kyoto Encyclopedia of Genes and Genomes (KEGG) pathway analysis further highlighted activation of multiple inflammatory and injury-related pathways, including complement and coagulation cascades, mRNA surveillance, and ECM-receptor interactions (Supplemental Figure 1B).

To identify key immune cell populations in disease progression, we analyzed single-cell RNA-seq (scRNA-seq) data from ex vivo–infected lung tissues of patients with COPD (14). Neutrophils emerged as the dominant immune population, with HMGB1 primarily expressed in alveolar epithelial cells (AT1 and AT2) and IL-33 highly enriched in neutrophils; NET-associated pathways were significantly activated (Figure 1, C and D). NET-associated pathways were significantly activated, and IL-33 was highly enriched in neutrophils (Figure 1, E and F). These findings were validated in independent scRNA-seq datasets from chemical-induced lung injury (GSE280922) (15) (Supplemental Figure 2, A–C) and COVID-19 pneumonia (GSE239835) (16) (Supplemental Figure 2, D–F), which consistently demonstrated enrichment of NET-related pathways and IL-33^+^ neutrophil accumulation (Supplemental Figure 2). Collectively, NET formation and neutrophil-derived IL-33 represent a conserved inflammatory signature across distinct lung injury etiologies.

### Elevated serum HMGB1, NET, and IL-33 levels are associated with SP-AKI in patients.

A multicenter cohort of 300 patients with severe pneumonia from 3 hospitals was enrolled to validate bioinformatics findings (Supplemental Figure 3 and Supplemental Table 1). The overall incidence of AKI was 53.6%, with 10.3% of patients requiring continuous renal replacement therapy (CRRT) and an in-hospital mortality rate of 14.6%. Compared with non-AKI patients, those with AKI exhibited significantly higher mortality (24.2% vs 3.6%, *P* < 0.001) and CRRT utilization rates (19.3% vs. 0.0%, *P* < 0.001). Although AKI incidence was higher in blood culture–positive patients, most AKI cases occurred in culture-negative patients, and blood culture status was not an independent risk factor on multivariable analysis (Supplemental Tables 1 and 2). Serum HMGB1, NET components (citrullinated histone H3–DNA [CitH3-DNA], neutrophil elastase–DNA [NE-DNA], and myeloperoxidase-DNA [MPO-DNA] complexes), IL-33, and neutrophil gelatinase-associated lipocalin (NGAL) were significantly elevated in patients with AKI, increased progressively with AKI severity, and positively correlated with each other and with renal function indices (Figure 1, G–N). Flow cytometry confirmed elevated IL-33 expression in circulating neutrophils from patients with SP-AKI (Figure 1, O and P).

To assess diagnostic performance, data from 1 center were divided into training and internal validation cohorts (70% and 30%, respectively), while patients from 2 additional hospitals constituted an external validation cohort (Supplemental Table 3). ROC analyses showed that NET markers and IL-33 consistently outperformed NGAL in discriminating SP-AKI. In the internal validation cohort, AUCs were 0.987 for IL-33, 0.958 for HMGB1, 0.914 for NE-DNA, 0.864 for MPO-DNA, and 0.822 for CitH3-DNA, versus 0.680 for NGAL. External validation yielded similar results, with NE-DNA achieving the highest AUC (0.974), followed by CitH3-DNA (0.948) and IL-33 (0.908) (Supplemental Figure 4 and Supplemental Tables 3 and 4). Optimal cutoff values derived from quartiles achieved clinically relevant sensitivity and specificity (Supplemental Table 4). Analysis of positive predictive value and negative predictive value across thresholds suggested that intermediate cutoffs were more suitable for identifying high-risk AKI patients, whereas lower or higher cutoffs favored AKI exclusion.

### HMGB1 upregulation induces neutrophil recruitment and NET formation in injured lungs.

**Figure 2:**
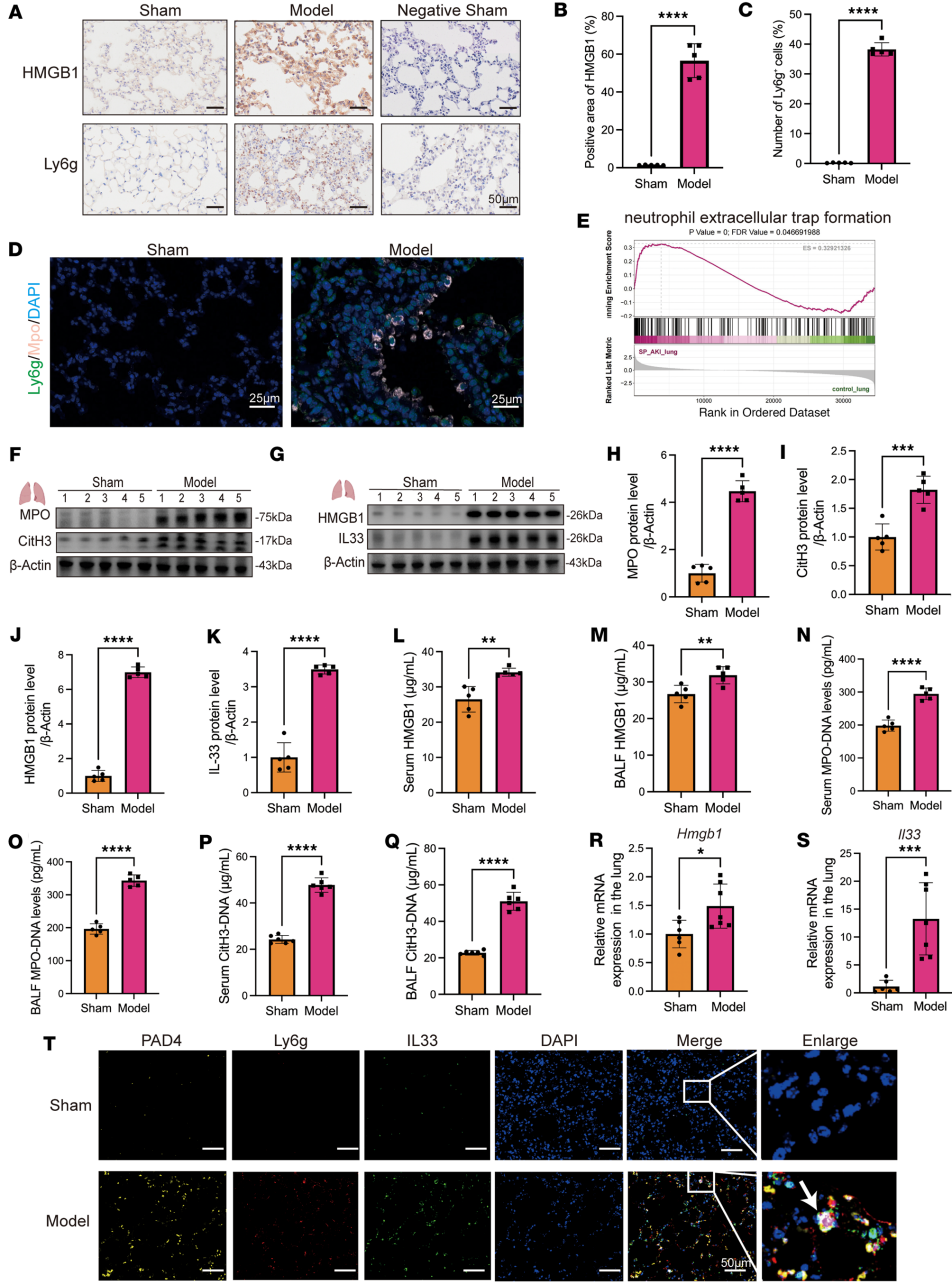
HMGB1 upregulation promotes neutrophil recruitment and IL-33–enriched NET formation in SP-AKI mouse lungs. (**A**) Representative immunohistochemical staining of HMGB1 and Ly6G in lung sections from sham and SP-AKI model mice. Negative staining controls are shown. Scale bars: 50 μm. (**B** and **C**) Quantification of HMGB1^+^ area and Ly6G^+^ neutrophils in lung sections from sham and SP-AKI model mice. (**D**) Representative immunofluorescence images showing Ly6G (green), MPO (pink), and DAPI (blue) staining in lung sections from sham and model mice. Scale bars: 25 μm. (**E**) Gene set enrichment analysis of RNA-seq data showing enrichment of the NET formation pathway in lung tissues from SP-AKI mice versus sham controls. (**F** and **G**) Western blots showing the expression of MPO, CitH3, HMGB1, and IL-33 in lung tissues. (**H**–**K**) Semiquantitative analysis of MPO (**H**), CitH3 (**I**), HMGB1(**J**), and IL-33 (**K**) protein levels. (**L**–**Q**) ELISA quantification of HMGB1, MPO-DNA complexes, and CitH3-DNA complexes in serum and BALF from sham and SP-AKI model mice. (**R** and **S**) Relative mRNA expression levels of HMGB1 (**R**) and IL-33 (**S**) in lung tissues. (**T**) Representative immunofluorescence images showing PAD4, Ly6G, and IL-33 expression in lung sections from sham and model mice, with merged and enlarged views. Scale bars: 50 μm. Enlarged panels show 5-fold magnification of boxed regions. Data are presented as mean ± SD. *n* = 5–7 per group. *P* values were calculated by 2-tailed Student’s *t* test. *P < 0.05, **P < 0.01, ***P < 0.001, ****P < 0.0001.

In patients with SP-AKI, serum HMGB1 levels positively correlated with NET markers, suggesting a potential role for HMGB1 in promoting NET formation (Figure 1N). To investigate the underlying mechanism, we established a murine SP-AKI model and focused on pulmonary HMGB1 dynamics. SP-AKI mice exhibited marked lung enlargement, pulmonary congestion, increased alveolar neutrophil infiltration, and thickened alveolar septa (Supplemental Figure 5, A–C). Lung RNA-seq analysis revealed significantly elevated HMGB1 expression accompanied by robust activation of innate immune responses, particularly pathways related to neutrophil chemotaxis, migration, and NET formation (Supplemental Figure 5, D–H). Consistently, HMGB1 expression was markedly upregulated in injured alveolar epithelial cells, as confirmed by immunofluorescence, Western blotting, and quantitative PCR (Figure 2, A, G, and R). This was accompanied by extensive neutrophil accumulation and pronounced NET deposition in lung tissues (Figure 2, A–K). Moreover, HMGB1 and NET components were significantly elevated in bronchoalveolar lavage fluid (BALF) and serum from SP-AKI mice (Figure 2, L–Q). Injured lungs also exhibited activation of NF-κB signaling and increased expression of proinflammatory cytokines, including IL-6, IL-1β, and TNF-α (Supplemental Figure 5, I–Q).

### NETs serve as systemic carriers of IL-33, preferentially driving renal injury.

**Figure 3:**
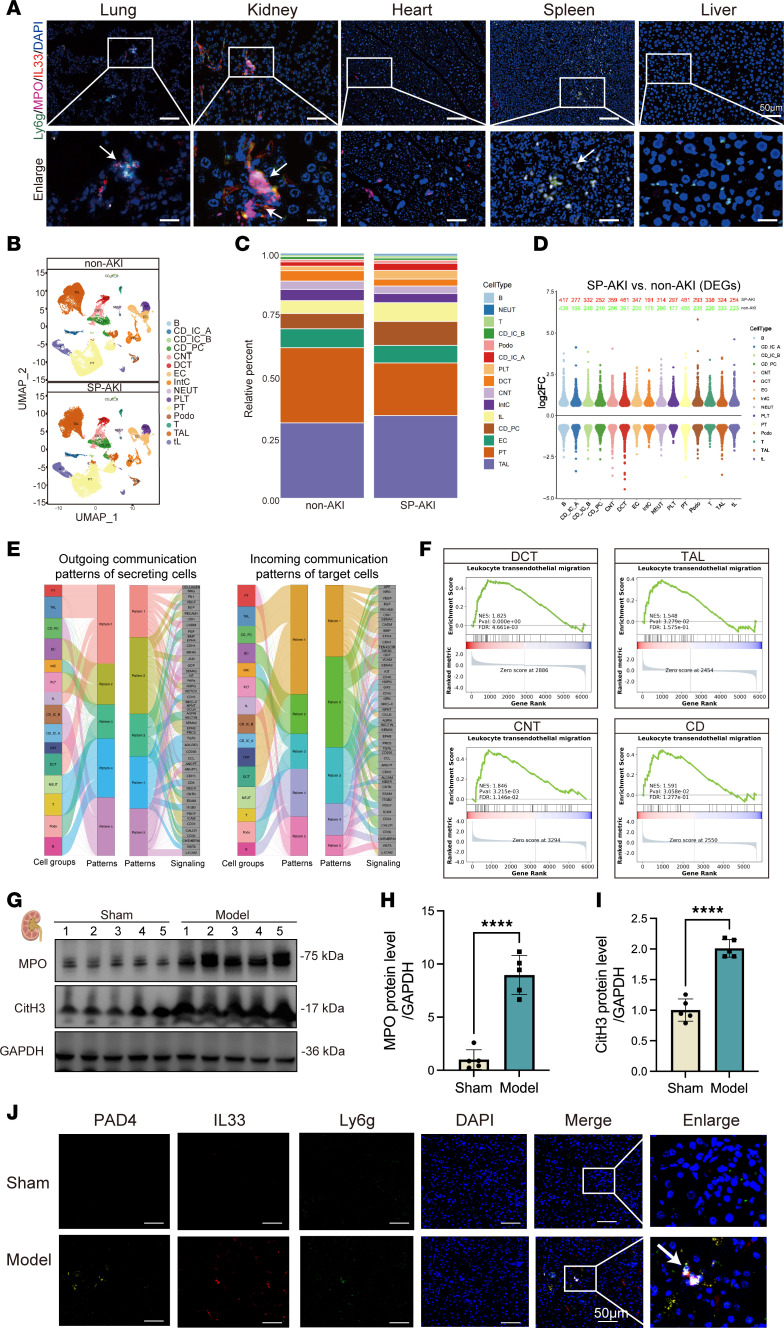
Accumulation of IL-33–enriched NETs in SP-AKI kidneys. (**A**) Representative confocal images of immunofluorescent staining of Ly6G (green), MPO (pink), IL-33 (red), and DAPI (blue) in the lung, kidney, heart, spleen, and liver from mice with severe pneumonia. Scale bars: 50 μm. The enlarged panels represent 3-fold magnification of the boxed regions. (**B**) Uniform manifold approximation and projection (UMAP) visualization of public scRNA-seq data showing major kidney cell populations in non-AKI and SP-AKI patients (GSE210622). (**C**) Relative proportions of major cell types in non-AKI and SP-AKI patient kidneys. (**D**) Violin plots showing differentially expressed genes (DEGs) across major kidney cell populations between SP-AKI and non-AKI conditions, presented as log_2_(fold change). (**E**) Cell-cell communication analysis showing outgoing signaling patterns of secreting cells (left) and incoming signaling patterns of target cells (right) in SP-AKI patient kidneys. (**F**) Gene set enrichment analysis of the leukocyte transendothelial migration pathway in distal convoluted tubule (DCT), thick ascending limb (TAL), connecting tubule (CNT), and collecting duct (CD). (**G**) Western blot showing the expression of MPO and CitH3 in SP-AKI mouse kidney. (**H** and **I**) Semiquantitative analysis of MPO (**H**) and CitH3 (**I**) protein levels. (**J**) Representative immunofluorescence images showing PAD4, IL-33, and Ly6G expression in SP-AKI mouse kidney sections from sham and model mice, with merged and enlarged views. Scale bars: 50 μm. Enlarged panels show 3-fold magnification of boxed regions. Data are presented as mean ± SD. *n* = 5 per group. *P* values were calculated by 2-tailed Student’s *t* test. ****P < 0.0001.

In injured lung tissues, quantitative PCR analysis revealed a marked upregulation of IL33 expression (Figure 2S). Consistently, immunofluorescence analysis demonstrated strong colocalization of IL-33 with NETs, marked by Ly6G and PAD4, indicating that IL-33 is incorporated into the NET scaffold rather than being passively released (Figure 2T). Given that NETs can disseminate systemically, we hypothesized that IL-33–bearing NETs act as carriers of remote organ injury. Multiplex tissue imaging revealed minimal NET deposition in the heart, liver, and spleen, whereas the kidney exhibited the most pronounced NET accumulation and associated tissue damage, identifying it as the primary target of systemic NET-mediated injury (Figure 3A).

This renal vulnerability likely reflects its dense vascular network and high filtration capacity, which increase exposure to circulating inflammatory mediators. Consistent with this model, transcriptomic analysis of SP-AKI mouse kidneys showed robust activation of innate immune pathways (Supplemental Figure 6, A–D). scRNA-seq of renal autopsy tissues from patients with severe pneumonia (GSE210622) further revealed peritubular neutrophil accumulation in the kidneys (Figure 3, B–F) (17). Furthermore, NETs were readily detected in SP-AKI mouse kidneys, where IL-33 colocalized with NETs, implicating IL-33–enriched NETs in amplifying renal inflammation (Figure 3, G–J).

### IL-33–abundant NETs from the lungs serve as key mediators of kidney damage.

**Figure 4:**
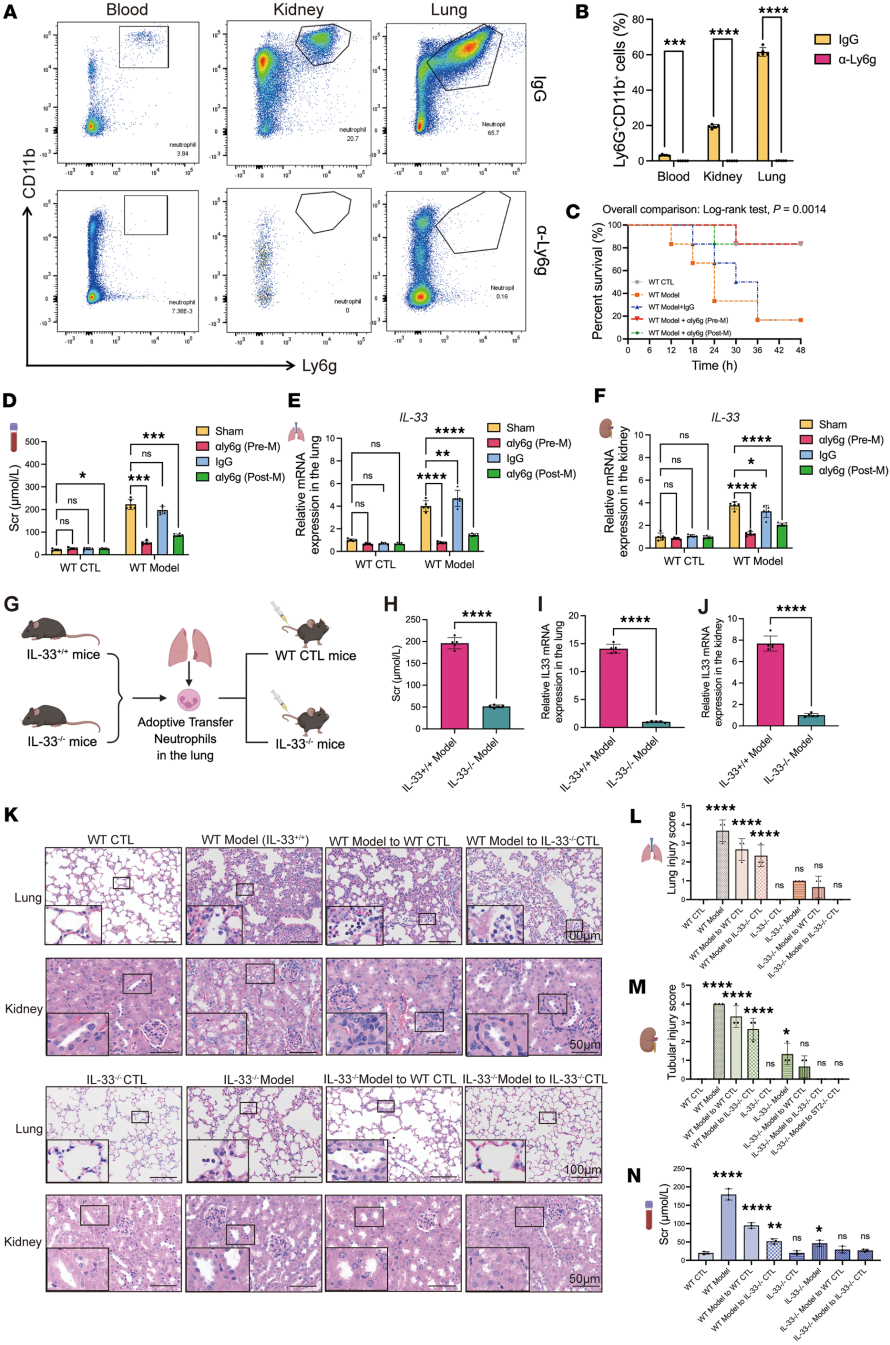
IL-33 from neutrophils is required for NET-driven lung and kidney injury in SP-AKI. (**A** and **B**) Representative flow cytometry plots and quantification of Ly6G^+^CD11b^+^ neutrophils in blood, kidney, and lung tissues following anti-Ly6G or IgG treatment. (**C**) Kaplan-Meier survival analysis of WT control mice, SP-AKI model mice, SP-AKI model mice treated with IgG, and SP-AKI model mice treated with anti-Ly6G antibody (250 μg/mouse) administered before (Pre-M) or after (Post-M) model induction. *P* value was determined by log-rank test. (**D**) Serum creatinine (Scr) levels in WT control and SP-AKI model mice treated with IgG, anti-Ly6G antibody Pre-M, or anti-Ly6G antibody Post-M. (**E** and **F**) Relative mRNA expression levels of IL-33 in lung (**E**) and kidney (**F**) tissues from each group. (**G**) Neutrophil adoptive transfer process in mouse lung tissues. IL-33^+/+^ mice representing the SP-AKI mouse model. All recipient mice for adoptive transfer underwent neutrophil depletion. (**H**) Scr levels were higher in IL-33^+/+^ model mice. (**I** and **J**) Relative IL-33 mRNA expression in lung (**I**) and kidney (**J**) tissues between IL-33^+/+^ and IL-33^–/–^ model. (**K**) Representative H&E-stained sections of lung and kidney tissues from WT and IL-33^–/–^ mice, as well as recipient mice following adoptive transfer of IL-33^+/+^ or IL-33^–/–^ model neutrophils. Scale bars: 100 μm (lung) and 50 μm (kidney). The enlarged panels show 3-fold magnification of the boxed regions in the lung images and 2-fold magnification in the kidney images. (**L** and **M**) Histological injury scores of lung (**L**) and kidney (**M**) tissues in the indicated groups. (**N**) Serum creatinine levels in WT and IL-33^–/–^ mice, as well as recipient mice following adoptive transfer of IL-33^+/+^ or IL-33^–/–^ model neutrophils. Data are presented as mean ± SD. *n* = 5 per group. *P* values were calculated by 2-way ANOVA with post hoc Holm-Šídák test (**B** and **D**–**F**), 2-tailed Student’s *t* test (**H**–**J**), or 1-way ANOVA (**L**–**N**). *P < 0.05; **P < 0.01; ***P < 0.001; ****P < 0.0001. NS, no statistically significant difference.

To validate the role of IL-33–enriched NETs in lung-kidney crosstalk, we performed in vivo neutrophil depletion using an anti-Ly6G antibody before and after SP-AKI induction. Neutrophil depletion effectively eliminated circulating and tissue-resident neutrophils, reduced IL-33 levels, attenuated lung and kidney injury, and significantly improved survival (Figure 4, A–F), demonstrating that IL-33 within NETs is a critical mediator of renal damage. To further confirm that IL-33–enriched NETs originating from injured lungs drive kidney injury, we isolated neutrophils from lung tissues of WT or IL-33–knockout (IL-33^–/–^) SP-AKI mice and adoptively transferred them into neutrophil-depleted WT recipients. Transfer of WT SP-AKI neutrophils restored lung and kidney injury, whereas IL-33^–/–^ neutrophils failed to do so. Moreover, transfer of WT SP-AKI neutrophils into otherwise-protected IL-33^–/–^ recipients reestablished significant organ damage (Figure 4, G–N).

Notably, previous studies have shown that neutrophils are short-lived innate immune cells, with a circulating half-life typically on the order of 6–8 hours (18). We found that neutrophils derived from injured lungs had a remarkably short half-life of less than 8 hours after adoptive transfer into the kidneys of recipient mice. In sharp contrast, neutrophils from healthy lungs remained detectable for over 24 hours after transfer. Despite their abbreviated survival, the injured lung–derived neutrophils exhibited significantly greater pathogenicity (Supplemental Figure 7).

### IL-33 drives renal tubular injury via ST2/NF-κB activation in vivo.

**Figure 5:**
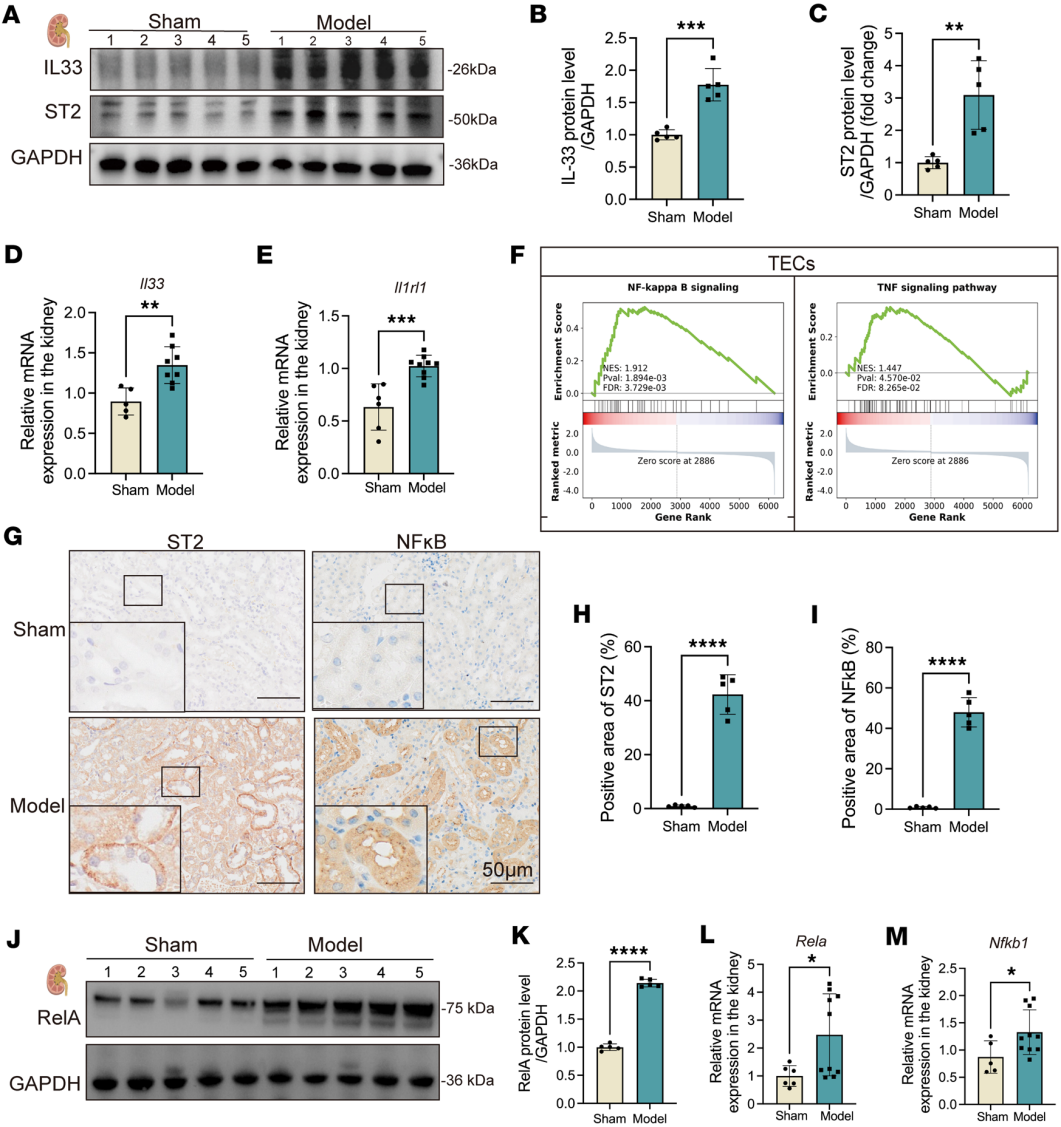
Activation of the IL-33/ST2/NF-κB signaling pathway in the kidneys during SP-AKI. (**A**) Western blot showing the expression of IL-33 and ST2 protein in kidney tissues from sham and SP-AKI model groups, with GAPDH as a loading control. (**B** and **C**) Semiquantitative analysis of IL-33 (**B**) and ST2 (**C**) protein levels. (**D** and **E**) Relative mRNA expression levels of IL-33 (**D**) and ST2 (**E**) in kidney tissues from sham and SP-AKI model mice. (**F**) Gene set enrichment analysis of a public scRNA-seq dataset (GSE210622) showing enrichment of the NF-κB and TNF signaling pathways in tubular epithelial cells (TECs). (**G**) Representative immunohistochemical staining of ST2 and NF-κB in kidney sections from sham and SP-AKI model mice. Scale bars: 50 μm. Enlarged panels show 3-fold magnification of boxed regions. (**H** and **I**) Quantification of ST2^+^ (**H**) and NF-κB^+^ (**I**) areas in kidney sections. (**J**) Western blot showing RelA (NF-κB subunit) expression in kidney tissues from sham and SP-AKI mice. (**K**) Semiquantitative analysis of RelA protein levels. (**L** and **M**) Relative mRNA expression levels of RelA (**L**) and NF-κB1 (**M**) in kidney tissues. Data are presented as mean ± SD. *n* = 5 per group. *P* values were calculated by 2-tailed Student’s *t* test. *P < 0.05, **P < 0.01, ***P < 0.001, ****P < 0.0001.

To investigate how IL-33–bearing NETs trigger kidney injury upon reaching the kidneys, we performed RNA-seq analysis of renal tissues from SP-AKI mice, which revealed pronounced enrichment of NF-κB, TNF-α, and IL-1β signaling pathways (Supplemental Figure 6). Elevated expression of ST2 and NF-κB components (RelA, P50/P105) in renal tissues suggested that IL-33 binds ST2 on tubular cells, activating downstream NF-κB signaling and driving proinflammatory cytokine transcription (IL-6, TNF-α), thereby exacerbating renal inflammation (Figure 5).

**Figure 6:**
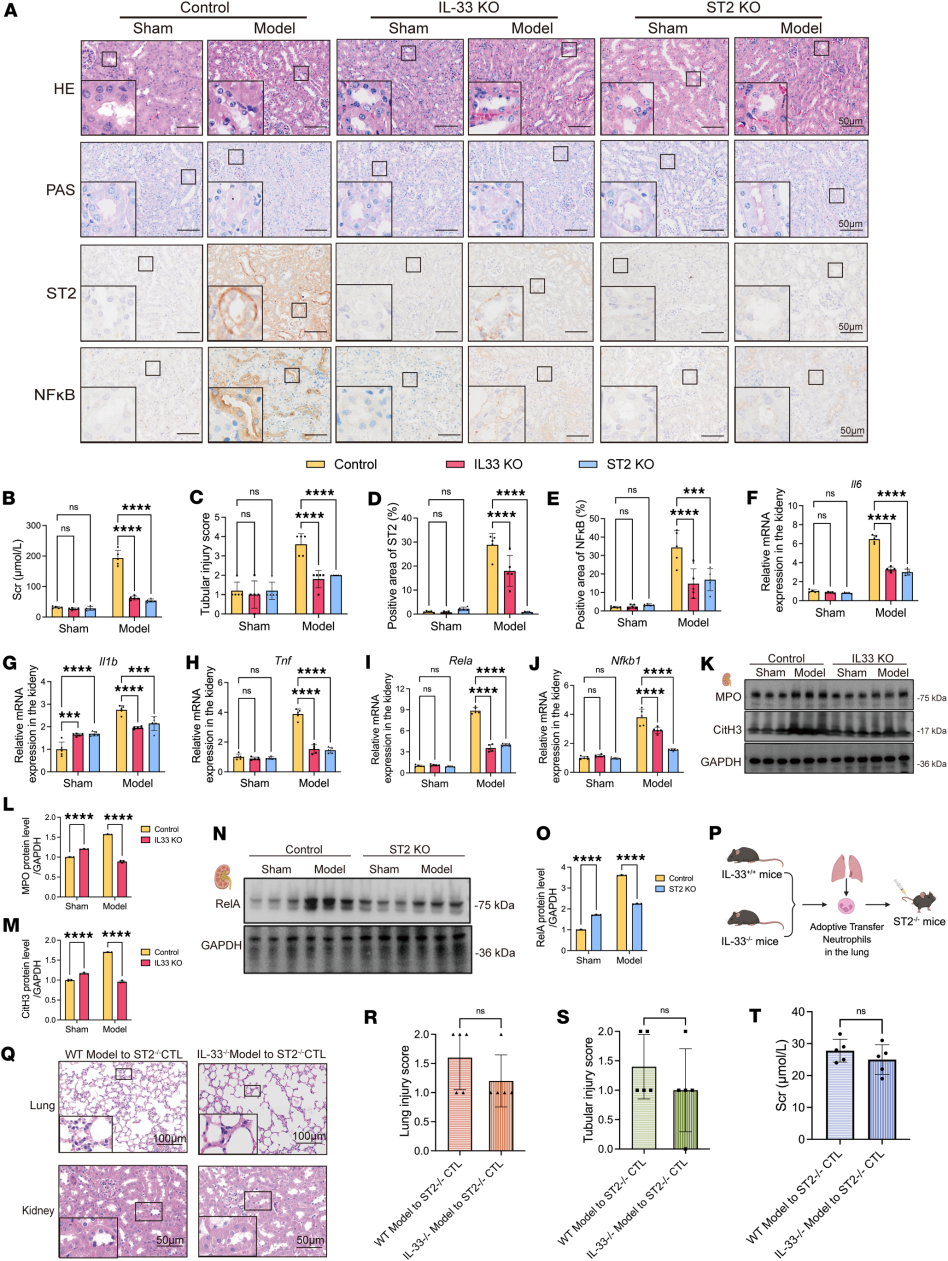
IL-33 and ST2 deficiency suppresses NET formation and NF-κB pathway activation in SP-AKI mice. (**A**) Representative H&E, periodic acid–Schiff (PAS), and immunohistochemical staining of ST2 and NF-κB in kidney sections from control, IL-33–knockout (IL-33 KO), and ST2-knockout (ST2 KO) mice under sham and SP-AKI model conditions. Scale bars: 50 μm. Enlarged panels show 4-fold magnification of boxed regions. (**B** and **C**) Serum creatinine (Scr) levels and renal tubular injury scores in each group. (**D** and **E**) Quantification of ST2^+^ (**D**) and NF-κB^+^ (**E**) areas in kidney sections. (**F**–**J**) Relative mRNA expression levels of IL-6 (**F**), IL-1β (**G**), TNF-α (**H**), RelA (**I**), and NF-κB1 (**J**) in kidney tissues of groups. (**K**) Western blot showing the expression of MPO and CitH3 in kidney tissues from control and IL-33 KO mice under sham and SP-AKI conditions, with GAPDH as a loading control. (**L** and **M**) Semiquantitative analysis of MPO (**L**) and CitH3 (**M**) protein levels. (**N**) Western blot showing the expression of RelA in kidney tissues from control and ST2 KO mice under sham and SP-AKI conditions, with GAPDH as a loading control. (**O**) Semiquantitative analysis of RelA protein levels. (**P**) Schematic illustration of adoptive transfer of neutrophils isolated from the lungs of IL-33^+/+^ or IL-33^–/–^ model mice into ST2^–/–^ recipient mice under SP-AKI conditions. (**Q**) Representative H&E-stained lung and kidney sections from ST2 KO mice receiving neutrophils from WT or IL-33^–/–^ mice. Scale bars: 100 μm (lung) and 50 μm (kidney). The enlarged panels represent 3-fold magnification in the lung images and 2-fold magnification in the kidney images. (**R**–**T**) Quantification of lung injury scores (**R**), renal tubular injury scores (**S**), and serum creatinine levels (**T**) in ST2 KO recipient mice following adoptive transfer. Data are presented as mean ± SD. *n* = 5 per group. *P* values were calculated by 2-way ANOVA with post hoc Holm-Šídák test (**B**–**J**, **L**, **M**, and **O**) or 2-tailed Student’s *t* test (**R** and **T**). ***P < 0.001; ****P < 0.0001. NS, no statistically significant difference.

To further elucidate the bridging role of the IL-33/ST2 axis in NET-induced renal inflammation, we established SP-AKI models in IL-33^–/–^ and ST2^–/–^ mice and found that both NET formation and activation of the downstream NF-κB inflammatory pathway were significantly suppressed (Figure 6, A–O). These data further illustrated that NETs disseminate to the kidneys, where they activate the NF-κB pathway through the IL-33/ST2 axis, resulting in the release of proinflammatory factors that contribute to renal injury. Disruption of IL-33/ST2 signaling mitigated multiorgan damage in SP-AKI by abrogating NET-mediated inflammation.

To determine whether this effect is mediated via the canonical IL-33/ST2 signaling axis, we adoptively transferred neutrophils from injured lung tissues isolated from WT or IL33^–/–^ SP-AKI mice into ST2^–/–^ recipients. Notably, ST2^–/–^ mice were protected from renal injury regardless of whether the transferred neutrophils were IL-33–competent or –deficient, demonstrating that IL-33 must signal through ST2 to exert its pathological effects (Figure 6, P–T).

Taken together, these results demonstrate that IL-33–enriched NETs formed in injured lung tissue disseminate to the kidneys and induce tubular epithelial cell damage via the IL-33/ST2/NF-κB signaling axis.

### HMGB1 amplifies renal inflammation by enhancing the NET/IL-33/ST2/NF-κB axis.

**Figure 7:**
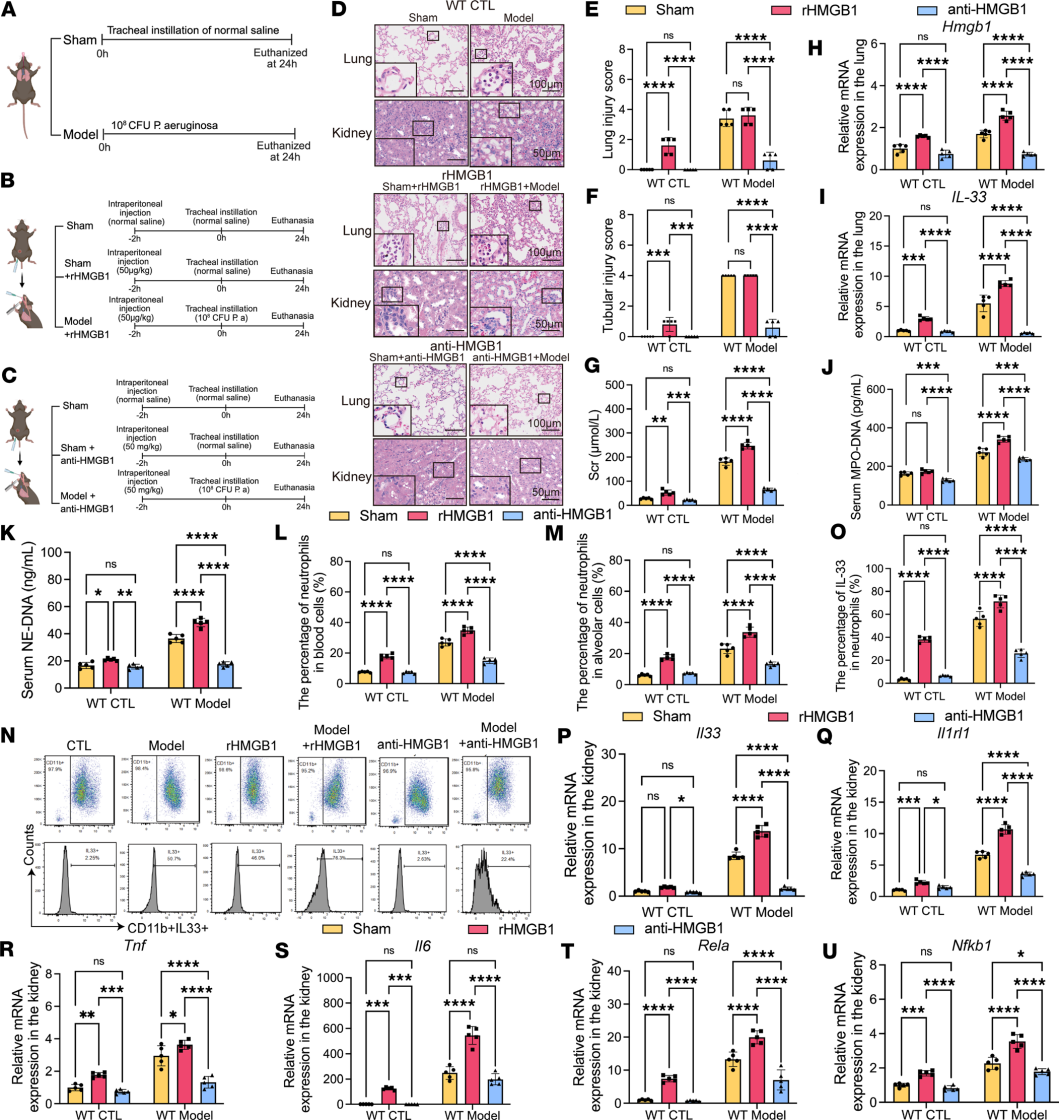
HMGB1 amplifies renal inflammation by enhancing the NET/IL-33/ST2/NF-κB axis. (**A**) Schematic illustration of the SP-AKI mouse model induced by intratracheal instillation of *Pseudomonas* *aeruginosa* or saline in WT mice. (**B**) Experimental design of recombinant HMGB1 (rHMGB1) administration (50 μg/kg) in sham and SP-AKI model mice. (**C**) Experimental design of HMGB1 neutralization using anti-HMGB1 antibody (glycyrrhizin, 50 mg/kg) in sham and SP-AKI model mice. (**D**) Representative H&E-stained sections of lung and kidney tissues from mice of different experimental groups. Scale bars: 100 μm (lung) and 50 μm (kidney). The enlarged panels show 3-fold magnification of the boxed regions in the lung images and 2-fold magnification in the kidney images. (**E**–**G**) Quantification of lung injury scores (**E**), renal tubular injury scores (**F**), and serum creatinine (Scr) levels (**G**) in WT control and SP-AKI model mice treated with rHMGB1 or anti-HMGB1 antibody. (**H** and **I**) Relative mRNA expression levels of HMGB1 (**H**) and IL-33 (**I**) in lung tissues from WT control and SP-AKI model mice following rHMGB1 administration or HMGB1 neutralization. (**J** and **K**) Serum MPO-DNA complex (**J**) and NE-DNA complex (**K**) levels in each group. (**L** and **M**) The percentage of neutrophils in whole blood cells (**L**) and BALF (**M**) of groups. (**N**) Representative flow cytometry plots showing CD11b^+^IL-33^+^ neutrophils in peripheral blood from the indicated groups. (**O**) Quantification of IL-33^+^ neutrophil percentages in groups from **N**. (**P**–**U**) Relative mRNA expression levels of IL-33 (**P**), ST2 (**Q**); inflammatory cytokines, including TNF-α (**R**) and IL-6 (**S**); and NF-κB pathway components, including RelA (**T**) and NF-κB1 (**U**), in kidney tissues from the indicated groups. Data are presented as mean ± SD. *n* = 5 per group. *P* values were calculated by 2-way ANOVA with post hoc Holm-Šídák test. *P < 0.05; **P < 0.01; ***P < 0.001; ****P < 0.0001. NS, no statistically significant difference.

To validate HMGB1’s role in promoting NETs and driving lung and kidney injury, we administered recombinant HMGB1 as an interventional challenge. Exogenous HMGB1 exacerbated kidney injury, increased IL-33 expression and NET formation in lung and kidney tissues, and activated ST2 and downstream NF-κB pathway markers in the kidneys. All effects were markedly reversed by glycyrrhizin, a functional HMGB1 inhibitor (Figure 7) (19), underscoring HMGB1’s pivotal role in driving the NET/IL-33/ST2/NF-κB axis.

Notably, this regulatory circuit is not unidirectional. Previous studies have shown that IL-33 can, in turn, activate HMGB1 release and exacerbate inflammatory responses (20). Consistent with this, we observed significantly reduced levels of HMGB1 and NET formation in the serum, BALF, and tissues of IL-33^–/–^ mice under injury conditions (Supplemental Figure 8).

Importantly, therapeutic targeting of HMGB1, IL-33, or NETs initiated 6 hours after SP-AKI induction still significantly reduced NET levels in the circulation and BALF (Supplemental Figure 9, A–K), and concomitantly suppressed ST2/NF-κB activation in lung and kidney tissues, with efficacy comparable to preemptive intervention (Supplemental Figure 9, L and M).

Collectively, these data reveal a self-amplifying feedback loop in SP-AKI whereby HMGB1 promotes IL-33–enriched NET formation, activates renal ST2/NF-κB signaling, and is further amplified by IL-33–driven HMGB1 release, identifying both HMGB1 and IL-33 as dual therapeutic targets.

### NET formation serves as a critical node in the HMGB1/IL-33 inflammatory loop.

**Figure 8:**
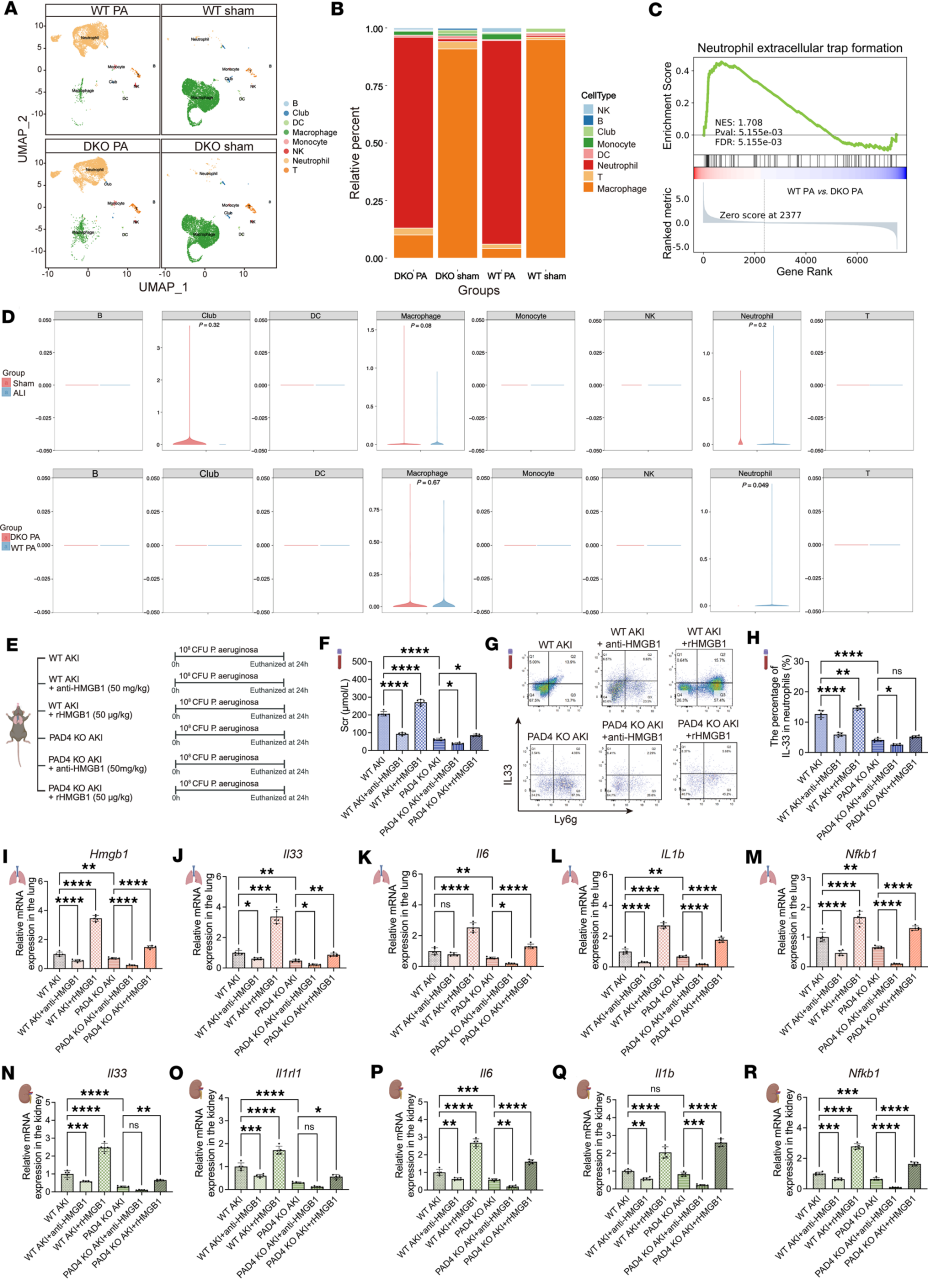
HMGB1 induces IL-33 release and aggravates SP-AKI through PAD4-dependent NET formation. (**A**) Uniform manifold approximation and projection (UMAP) visualization of public scRNA-seq data (GSE274823) derived from BALF in a murine model of acute lung injury (ALI) induced by intratracheal *Pseudomonas* *aeruginosa* (PA) infection. Comparative analysis between WT and PAD4/PAD2 double-knockout (DKO) mice reveals distinct cellular subpopulations. Each point represents a single cell, and clusters were identified based on gene expression profiles. Cell types are color-coded as indicated in the legend. (**B**) Stacked bar plot depicting the relative abundance of major cell types in BALF across each group. (**C**) Gene set enrichment analysis of the NET formation pathway (predominantly enriched in immature neutrophils) in BALF. (**D**) The expression levels of the IL-33 gene among various cell types across different groups. (**E**) Experimental design illustrating WT and PAD4-knockout (PAD4 KO) mice subjected to SP-AKI and treated with recombinant HMGB1 (rHMGB1, 50 μg/kg) or anti-HMGB1 antibody (glycyrrhizin, 50 mg/kg). (**F**) Serum creatinine (Scr) levels in WT and PAD4 KO mice with SP-AKI following rHMGB1 administration or HMGB1 neutralization. (**G** and **H**) Flow cytometry and quantitative analysis of IL-33^+^ in neutrophils of blood across each group. (**I**–**M**) Relative mRNA expression levels of HMGB1 (**I**), IL-33 (**J**), inflammatory cytokines IL-6 (**K**) and IL-1β (**L**), and NF-κB1 (**M**) in lung tissues from the indicated groups. (**N**–**R**) Relative mRNA expression levels of IL-33 (**N**), ST2 (**O**), inflammatory cytokines IL-6 (**P**) and IL-1β (**Q**), and NF-κB1 (**R**), in kidney tissues from the indicated groups. Data are presented as mean ± SD. *n* = 5 per group. *P* values were calculated by 1-way ANOVA with post hoc Holm-Šídák test. *P < 0.05; **P < 0.01; ***P < 0.001; ****P < 0.0001. NS, no statistically significant difference.

To determine whether NETs serve as a major source of IL-33, we analyzed scRNA-seq data from BALF of acute lung injury mice infected with *Pseudomonas* *aeruginosa* (PA) (GSE274823) (21). PA infection induced marked neutrophil recruitment, with IL-33 predominantly expressed by neutrophils. In PAD4/PAD2 double-knockout mice, although neutrophil infiltration persisted, NET formation was markedly reduced, and neutrophil IL-33 expression was nearly absent (Figure 8, A–D). Further analysis of immune cells from blood, lungs, and kidneys of WT sham, WT SP-AKI, IL-33^–/–^ SP-AKI, and PAD4^–/–^ SP-AKI mice confirmed injured neutrophils as the major source of IL-33 (Supplemental Figures 10–12). Importantly, PAD4 deficiency significantly reduced neutrophil-derived IL-33 release and alleviated kidney injury, indicating that neutrophil-derived IL-33 is dependent on NET formation (Figure 8, E–H).

Moreover, PAD4 deficiency disrupted the HMGB1/NET/IL-33 inflammatory circuit and blunted the associated cytokine storm, as administration of exogenous recombinant HMGB1 failed to induce IL-33 overproduction. Notably, PAD4 deletion also abolished activation of the IL-33/ST2/NF-κB signaling pathway in the kidney (Figure 8, I–R). These findings establish NETs as both effector mediators and inflammatory amplifiers in SP-AKI lung-kidney crosstalk.

### Anti-HMGB1 and anti–IL-33 interventions attenuate NET formation and tubular injury in vitro.

**Figure 9:**
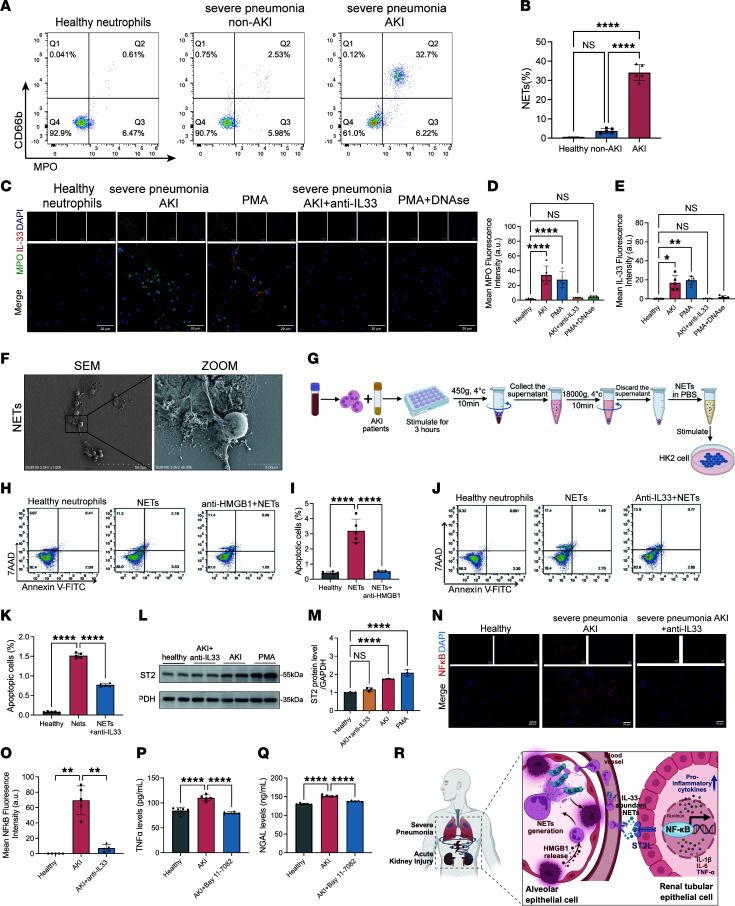
HMGB1/IL-33 neutralization reduces NETs and renal tubular damage in vitro. (**A** and **B**) Representative flow cytometry plots and quantification of NET formation in neutrophils from healthy donors, severe pneumonia patients without AKI, and SP-AKI patients, gated as CD66b^+^MPO^+^. (**C**) Immunofluorescence showing that IL-33 colocalized with MPO within NET structures. Pretreatment with anti–IL-33 (1 μg/mL) reduced the formation of NETs. Scale bars: 20 μm. (**D** and **E**) Quantification of mean fluorescence intensity of MPO (**D**) and IL-33 (**E**) in neutrophils under the indicated conditions. (**F**) Scanning electron microscopy showing the morphology of NETs after neutrophils were stimulated by serum from patients with SP-AKI. Scale bars: 50 μm (left) and 5 μm (right). (**G**) Schematic illustration of NET isolation from SP-AKI neutrophils and subsequent stimulation of HK-2 cells. (**H**) Flow cytometric analysis of apoptosis in HK-2 cells cocultured with healthy neutrophils, NETs, and anti-HMGB1–treated (glycyrrhizic acid, 200 μM) NETs. (**I**) Inhibition of NET-induced HK-2 cell apoptosis after anti-HMGB1 administration. (**J**) Flow cytometric analysis of apoptosis in HK-2 cells cocultured with healthy neutrophils, NETs, and anti–IL-33–treated NETs. (**K**) Inhibition of NET-induced HK-2 cell apoptosis after anti–IL-33 administration. (**L** and **M**) Western blot analysis and quantification of ST2 expression in HK-2 cells after stimulation with NETs from healthy donors, patients with SP-AKI, anti–IL-33–treated SP-AKI neutrophils, or PMA-treated neutrophils. (**N** and **O**) Immunofluorescence analysis of NF-κB activation in HK-2 cells following NET stimulation and quantification of fluorescence intensity. Scale bars: 20 μm. (**P** and **Q**) ELISA measurement of TNF-α and NGAL levels in cell culture supernatants following pretreatment with the NF-κB inhibitor Bay 11-7082 (2 μM). (**R**) Mechanisms of SP-AKI. Data are presented as mean ± SD. *n* = 5 per group. *P* values were calculated by 1-way ANOVA with post hoc Holm-Šídák test. *P < 0.05; **P < 0.01; ****P < 0.0001. NS, no statistically significant difference.

To further investigate the pathogenic roles of HMGB1 and IL-33 in NET-mediated tubular injury, we established an in vitro model mimicking the inflammatory microenvironment of SP-AKI. Serum from blood culture–negative patients with severe pneumonia was used to stimulate neutrophils from healthy donors in vitro. Flow cytometric analysis revealed a marked NET increase, characterized by coexpression of CD66b and MPO, especially in those who progressed to SP-AKI (Figure 9, A and B). Scanning electron microscopy showed typical NET morphology, including neutrophil membrane rupture and extensive extracellular chromatin fiber release (Figure 9F).

Coculture of patient serum–induced NETs with HK-2 cells caused significant tubular cell apoptosis, which was markedly attenuated by glycyrrhizin, implicating HMGB1 as a key mediator (Figure 9, G–I). Immunofluorescence analysis confirmed IL-33 incorporation into NETs, colocalizing with MPO, identifying IL-33 as both a structural and functional NET component; anti–IL-33 neutralization significantly suppressed NET formation and HK-2 apoptosis (Figure 9, C–E, J, and K).

Western blot and immunofluorescence analyses demonstrated that NET stimulation upregulated ST2 and activated NF-κB signaling in HK-2 cells, both of which were effectively reversed by anti–IL-33 treatment, reinforcing the pivotal role of IL-33/ST2/NF-κB signaling in NET-induced tubular injury (Figure 9, L–O). Consistent with this, pretreatment with NF-κB inhibitor Bay 11-7082 significantly alleviated NET-induced cytotoxicity, reducing NGAL levels and TNF-α secretion (Figure 9, P and Q).

Together, these findings strongly support the conclusion that NETs contribute to renal tubular injury through the IL-33/ST2/NF-κB axis and highlight the therapeutic potential of targeting HMGB1 or IL-33 to mitigate NET-associated renal damage in SP-AKI.

The proposed mechanism underlying SP-AKI is summarized in Figure 9R. Upon PAMP exposure, the lung mounts an immune response, releasing DAMPs such as HMGB1, NETs, and IL-33. HMGB1 promotes NET formation and IL-33 release, while IL-33 further damages epithelial cells, amplifying HMGB1 release — forming a self-sustaining HMGB1/NET/IL-33 loop. Notably, the release of IL-33 is dependent on HMGB1-induced NET activation. Circulating IL-33–enriched NETs drive systemic inflammation and, upon reaching the kidney, activate ST2/NF-κB signaling in tubular epithelial cells, causing inflammation and injury.

## Discussion

SP-AKI is common and highly lethal, yet the mechanisms underlying lung-kidney inflammatory crosstalk remain incompletely defined. By integrating a multicenter clinical cohort with multiomics analyses and complementary genetic and pharmacological approaches in murine models, our study proposes and validates a DAMP-driven HMGB1/NET/IL-33 lung-kidney inflammatory axis, providing a mechanistic framework to explain the initiation and propagation of remote renal injury in the setting of severe pneumonia.

In our multicenter cohort, we found that circulating levels of HMGB1, NET-related markers, and IL-33 were significantly associated with both the occurrence and severity of SP-AKI, and notably, these associations remained robust in patients with negative blood cultures. These findings suggest that the drivers of renal injury are not determined solely by PAMPs; rather, sterile inflammation and DAMPs may occupy a central role in the pathogenesis of SP-AKI. This concept is consistent with clinical observations that, in patients with severe pneumonia, antimicrobial therapy alone is often insufficient to control immune-mediated organ injury (22). In contrast, therapeutic strategies targeting key inflammatory mediators, such as cytokine-neutralizing approaches, may hold greater promise by interrupting inflammatory cascades in both local and remote organs, thereby improving overall clinical outcomes.

In addition, we observed a consistent enrichment of NETs and IL-33^+^ neutrophils across multiple models of lung injury, indicating that this phenotype may represent a shared inflammatory hallmark of pulmonary damage. Given the established role of NETs in distant organ injury, we proposed that neutrophil-derived IL-33, released via NETs, could be a key mediator of renal damage secondary to lung injury. Previous studies have highlighted the pathogenic role of NETs in kidney injury, particularly in AKI (8, 23). The entry of circulating NETs into the kidney and their contribution to tubular damage likely involve multiple mechanisms, including their retention within the renal microvasculature (24), receptor-mediated uptake of NET-derived fragments by proximal tubular epithelial cells with ensuing inflammatory activation (25), chemokine-driven amplification of neutrophil recruitment and NET formation (26), and the direct cytotoxic effects of NETs following their translocation across the tubular barrier (27). This study primarily focuses on the direct damaging effects on renal tubular cells exerted by inflammatory mediators, specifically IL-33, released from NETs.

Notably, although IL-33 can be expressed by multiple cell types, its sustained upregulation in neutrophils under injurious conditions was consistently observed across different experimental models. On this basis, we further focused on the mode of release and the biologically active form of neutrophil-derived IL-33 to elucidate its mechanistic role in kidney injury secondary to lung injury. As a prototypical alarmin cytokine, IL-33 binds to its receptor ST2L and activates NF-κB signaling, thereby promoting the production of proinflammatory cytokines and amplifying inflammatory responses (28). A key question, however, is how posttranslational modifications — particularly proteolytic processing and oxidative modification — shape the biological activity of IL-33. In the specific microenvironment of NET formation, in what form does IL-33 exist and exert its function?

In the present study, IL-33 was predominantly released during NET formation, a process characterized by nuclear envelope disruption and a protease-rich milieu. Under such conditions, IL-33 is more likely to undergo proteolytic processing rather than oxidative inactivation. Consistent with this notion, previous studies have demonstrated that neutrophil-derived proteases, including NE, cathepsin G, and proteinase 3, cleave full-length IL-33 into shorter mature forms with enhanced biological activity (29). By contrast, although oxidative modification has been shown to rapidly inactivate extracellular IL-33 by disrupting its interaction with ST2 (30), this process is more likely to occur in oxygen-rich extracellular environments and may be less prominent in the protease-dense milieu associated with NET formation. Collectively, these lines of evidence suggest that the IL-33 detected in our study predominantly represents a protease-processed, biologically active form released during NET formation, rather than an oxidatively inactivated, functionally inert species. Moreover, our findings that renal ST2 signaling is responsive to IL-33 provide functional support for the notion that the IL-33 identified in this study is present largely in its bioactive form.

In addition, our study further substantiates the critical role of the DAMP HMGB1 in driving NET formation and amplifying inflammatory responses. Our previous work demonstrated that, in patients with severe pneumonia requiring extracorporeal life support, mechanical ventilation–associated HMGB1 activation markedly promotes NET generation (31). These findings are consistent with multiple previous studies showing that HMGB1 induces NET formation, whereas its antagonists effectively suppress this process (9, 32, 33). Accordingly, targeting HMGB1 has been proposed as a potential therapeutic strategy to reduce the incidence and mortality of SP-AKI (34).

Building on this framework, we further delineated a bidirectional regulatory relationship between HMGB1 and IL-33. We found that exogenous HMGB1 or its specific inhibitor respectively enhanced or suppressed IL-33 release, in line with previous reports describing an intrinsic IL-33 auto-amplification loop driven by HMGB1 (35–37). Importantly, we observed that genetic deletion of IL-33 not only reduced IL-33 levels but also significantly attenuated HMGB1 release and NET formation. These findings suggest that IL-33 functions not only as a structural component of NETs but also as a DAMP that feeds back to promote HMGB1 release from injured epithelial cells, thereby establishing a self-amplifying HMGB1/NET/IL-33 positive-feedback loop.

Notably, under conditions of PAD4 deficiency, which specifically abrogates NET formation, exogenous HMGB1 stimulation failed to effectively induce IL-33 release. This result indicates that NET formation is a critical prerequisite for the establishment of the HMGB1/IL-33 inflammatory circuit. In this context, NETs do not merely act as downstream effector structures that amplify inflammation but rather serve as a central signaling bridge linking HMGB1 to IL-33. These genetic data further strengthen our proposed mechanistic model in which HMGB1 drives NET formation to facilitate the functional release of IL-33, while IL-33 in turn augments HMGB1-associated inflammatory responses, together forming a self-sustaining DAMP-driven inflammatory loop. This feedback circuit persistently fuels inflammatory responses and tissue injury during the development of SP-AKI, underscoring the central role of this signaling axis in disease pathogenesis. More importantly, in the present study, targeting HMGB1, IL-33, or NETs — in both preventive and therapeutic settings — consistently conferred significant protective effects, further supporting the translational potential of this pathway as a promising therapeutic target in SP-AKI.

This study redefines NETs from mere local inflammatory effectors to cross-organ pathogenic carriers that link lung injury to remote kidney damage. Through integrated genetic and pharmacological evidence, we establish a causal role for the HMGB1/NET/IL-33 axis in SP-AKI, providing a mechanistically defined paradigm for interorgan inflammatory injury and laying a theoretical foundation for therapeutic strategies targeting DAMPs. We also recognize that lung-kidney interactions are orchestrated by a complex and multilayered regulatory network that extends beyond the HMGB1/NET/IL-33 axis examined here. In addition to humoral inflammatory mediators, accumulating evidence indicates that neuroimmune pathways play an important role in interorgan communication, including the regulation of inflammation and organ function by the autonomic nervous system (38, 39). However, the present study was specifically designed to delineate DAMP-driven humoral signaling mechanisms, and therefore did not address the potential contributions of neuroimmune circuits or other non-soluble regulatory pathways. Moreover, although our data support a pathogenic role for neutrophil-derived IL-33 in NET-mediated renal injury, future studies employing neutrophil-specific IL-33–deficient models will be required to definitively establish its causal contribution to the lung-kidney inflammatory axis.

In summary, we showed that lung injury–derived HMGB1 drove the formation of IL-33–enriched NETs, which reached the kidney and exacerbated tubular inflammation through the IL-33/ST2/NF-κB axis, thereby promoting SP-AKI. These findings not only fill a critical mechanistic gap in our understanding of pneumonia-associated secondary kidney injury but also provide a translational framework for therapeutic strategies targeting the HMGB1/NET/IL-33 axis.

## Methods

Further information can be found in Supplemental Methods. All oligonucleotide primers used in this study are provided in Supplemental Table 5, and all antibodies are listed in Supplemental Tables 6 and 7.

### Sex as a biological variable.

For human studies, samples from both male and female participants were included (Supplemental Table 1), and no sex-specific differences were observed in the analyzed outcomes. In animal experiments, only male mice were used, as previous studies have reported that male mice develop more severe pneumonia following infection compared with female mice (40, 41).

### Study design and population.

We conducted a prospective, multicenter cohort study at Sir Run Run Hospital of Nanjing Medical University, Nanjing First Hospital, and Wuxi People’s Hospital Affiliated with Nanjing Medical University. The study enrolled adult patients (≥18 years) diagnosed with severe pneumonia between January, 2022 and September, 2023. Exclusion criteria included (a) hospital stay less than 48 hours or incomplete clinical data; (b) less than 2 serum creatinine measurements during hospitalization; (c) end-stage renal disease requiring maintenance dialysis; and (d) AKI caused by nonpulmonary factors, previous nephrotoxic agent exposure, or contrast administration before admission.

### Construction of SP-AKI mouse model.

Male C57BL/6 mice (8–10 weeks old, 20–25 g) were purchased from the Model Animal Research Center of Nanjing University, while IL-33^–/–^ and ST2^–/–^ mice were provided by Zhang Hongsong’s team (Nanjing First Hospital). PAD4^–/–^ mice were purchased from Cyagen Biosciences Inc. Mice were anesthetized by inhalation of isoflurane, and 50 μL of PA suspension (1 × 10^8^ CFU) was intratracheally inoculated to establish SP-AKI; sham received 50 μL sterile saline. Mice were euthanized at 24 hours after infection, and blood, BALF, lung, and kidney tissues were collected for analysis.

### Experimental cell sources and culture.

HK-2 cells were obtained from Shanghai Zhongqiao Xinzhou Biotechnology Co., Ltd. Neutrophils were cultured in RPMI-1640 medium supplemented with 10% fetal bovine serum, while HK-2 cells were maintained in DMEM/F12 medium with 10% fetal bovine serum. All cells were incubated at 37°C in a humidified atmosphere containing 5% CO_2_.

### Intervention with anti–IL-33, anti-HMGB1, anti–NF-κB, and recombinant mouse HMGB1.

In vitro, neutrophils were pretreated with anti–IL-33 neutralizing antibody (1 μg/mL; R&D Systems) for 4 hours; SP-AKI patient serum was incubated with glycyrrhizic acid (200 μM; MedChemExpress) for 24 hours; and HK-2 cells were pretreated with Bay 11-7082 (2 μM; Sigma-Aldrich) for 24 hours. In vivo, mice received intraperitoneal injections of recombinant HMGB1 (50 μg/kg; Sino Biological), glycyrrhizic acid (50 mg/kg; MedChemExpress), anti-HMGB1 antibody (100 μg/kg; Arigo), anti–IL-33 antibody (60 μg/kg; R&D Systems), or GSK484 (4 mg/kg; MedChemExpress); controls received equivalent volumes of sterile saline.

### Statistics.

Normality was assessed using the Shapiro-Wilk test (<50 samples) or Kolmogorov-Smirnov test (≥50 samples). Normally distributed data are presented as mean ± SD and were analyzed by 2-tailed Student’s *t* test; non–normally distributed data are presented as median (IQR) and were analyzed using the Mann-Whitney *U* test. Multiple-group comparisons used 1-way or 2-way ANOVA with post hoc tests. Categorical variables were analyzed by χ^2^ test. Analyses were performed using SPSS 22.0 (IBM), GraphPad Prism 9.5.1, and R 4.2.2 (https://www.r-project.org/). Statistical significance was set at a *P* value of less than 0.05.

### Study approval.

All animal experiments were approved by the Institutional Animal Care and Use Committee (protocol number: IACUC-2311001) and conducted in accordance with the institutional guidelines of Nanjing Medical University for animal experimentation. The human study was performed in accordance with the Declaration of Helsinki and approved by the Institutional Review Boards of Sir Run Run Hospital (2021-SR-042), Nanjing First Hospital (KY20230410-01-KS-01), and Wuxi People’s Hospital (KY22073), Nanjing Medical University. Written informed consent was obtained from all participants.

### Data availability.

All the data generated or analyzed in our study are available as supplemental files. Bulk RNA-seq data generated in this study are deposited in the NCBI Gene Expression Omnibus (GEO) database under accession number GSE324794; proteomics data are available in the OMIX database under accession number OMIX015195. Values for all data points in graphs are reported in the Supporting Data Values file. Clinical data used in this study involve human participants and are not publicly available due to privacy and ethical restrictions.

## Author contributions

C Cao and XW designed the study. MM and HZ conceived the study, interpreted data, and drafted the manuscript. WD, XD, MC, DC, BP, C Chen, TC, and ZW participated in the design of the study and acquired data. MM and HZ contributed equally to this work. The order of co–first authors was determined based on their relative contributions to the study. All authors revised the manuscript and approved the final version of the manuscript.

## Conflict of interest

The authors have declared that no conflict of interest exists.

## Funding support

- National Natural Science Foundation of China (82170698 and 82500834).
- Jiangsu Funding Program for Excellent Postdoctoral (2025ZB004).
- The Major Project of Jiangsu Provincial Health Commission Medical Research (ZDB2020023).
- The General Project of Jiangsu Provincial Health Commission Medical Research (H2023057).
- Jiangsu Provincial Social Development Biomedical Research Fund (BE2023830).
- Nanjing Municipal Health Science and Technology Development Special Fund Key Project (ZKX22035).

## Supplementary Material

Supplemental data

Unedited blot and gel images

Supporting data values

## Version 1 05/22/2026

Electronic publication
