# Supplementary material for: HMGB1-mediated formation of IL-33–abundant NETs drives lung-to-kidney injury in severe pneumonia–associated acute kidney injury
Source: JCI Insight. 2026 May 22;11(10):e191979. doi: 10.1172/jci.insight.191979 (PMC13232728; doi:10.1172/jci.insight.191979)
Supplement: Supplemental data [file jciinsight-11-191979-s286.pdf]

Supplementary Material

Supplementary File (PDF)

Supplementary Methods

Supplemental Figure 1. Proteomics-identified signaling pathways associated with SP-AKI in patient serum

Supplemental Figure 2. Public scRNA-seq analyses reveal IL33+neutrophil enrichment and activation of NETs-related pathways in different lung injury

Supplemental Figure 3. Flowchart of patient enrollment in the severe pneumonia–acute kidney injury (SP-AKI) cohort.

Supplemental Figure 4. Discriminative ability of circulating biomarkers for SP-AKI.

Supplemental Figure 5. RNA sequencing analysis of lung tissues in SP-AKI mouse model.

Supplemental Figure 6. RNA sequencing analysis of kidney tissues in SP-AKI mouse model.

Supplemental Figure 7. In vivo half-life of adoptively transferred neutrophils in recipient mice and their temporal impact on kidney injury

Supplemental Figure 8. IL-33 deficiency reduced HMGB1 release and NETs formation.

Supplemental Figure 9. Therapeutic effects of targeting IL-33/HMGB1/NETs in SP-AKI.

Supplemental Figure 10. Flow cytometric analysis of IL-33 expression in circulating immune cell subsets across experimental groups.

Supplemental Figure 11. Flow cytometric analysis of IL-33 expression in kidney immune cell subsets across experimental groups.

Supplemental Figure 12. Flow cytometric analysis of IL-33 expression in lung immune cell subsets across experimental groups.

Supplemental Table 1. Comparison of clinical data between SP-AKI and SP-non-AKI patients in severe pneumonia.

Supplemental Table 2. Multivariate logistic analysis of independent risk factors for SP-AKI.

Supplemental Table 3. Expression levels of each biomarker in the three datasets.

Supplemental Table 4. Sensitivity and specificity of biomarkers at different cutoff values.

Supplemental Table 5. Primer sequences used in this study.

Supplemental Table 6. Specific primary antibodies used in this study.

Supplemental Table 7. Antibodies used for flow cytometry.

Supplementary Methods

Definitions

Acute kidney injury (AKI) was defined according to Kidney Disease: Improving Global Outcomes (KDIGO) criteria as an increase in serum creatinine (sCr) ≥26.5 μmol/L within 48 h or ≥1.5-fold from baseline within 7 days (1). Severe AKI was defined as KDIGO stage 2–3, including sCr ≥2-fold from baseline or ≥3.0 mg/dL (≥265 μmol/L), oliguria (<0.5 mL/kg/h for ≥12 h), or stage 3 criteria (sCr ≥4.0 mg/dL [≥354 μmol/L] or initiation of renal replacement therapy). Baseline sCr was defined as the lowest value recorded during hospitalization. The definition of severe pneumonia referred to the diagnostic criteria for severe pneumonia in the 2016 edition of the "Guidelines for the Diagnosis and Treatment of Community-Acquired Pneumonia in Adults in China"(2). Major criteria: requirement for endotracheal intubation and mechanical ventilation or septic shock requiring vasopressor support after active fluid resuscitation. Minor criteria: respiratory rate ≥ to 30 times/min; oxygenation index ≤ 250 mmHg; multi-lobar infiltration; impaired consciousness and/or disorientation; blood urea nitrogen ≥ 7.14 mmol/L; systolic blood pressure < 90 mmHg despite active fluid resuscitation. Severe pneumonia was diagnosed if any one of the above major criteria or at least three of the minor criteria were met.

Outcomes

The primary endpoint of this study was AKI. Secondary outcomes included continuous renal replacement therapy (CRRT) use and in-hospital death. Patients were categorized into SP-AKI and SP-nonAKI groups based on SP-AKI occurrence.

Sample size calculation

The sample size was calculated using SPASS software, assuming a diagnostic sensitivity and specificity of 80% (minimum acceptable thresholds: 60%). With an allowable error of 20% for sensitivity and 10% for specificity, and an expected AKI incidence of 60%, the minimum required sample size was 142 patients. A total of 300 patients were included in this study, meeting the minimum inclusion criteria.

Collection of clinical data

The clinical data of patients were collected, including gender, age, past medical history, laboratory test indicators, and intervention measures etc.

Clinical sample collection

Peripheral blood specimens were collected upon admission. After collection, blood samples were centrifuged at 3,000*g* for 10 min at 4°C. The supernatant was transferred to microcentrifuge tubes and immediately stored at -80°C until further analysis.

Clinical sample testing

Serum levels of human or mouse NGAL, CitH3–DNA complexes, MPO–DNA complexes, NE–DNA complexes, IL-33, and HMGB1 were quantified using commercially available human or mouse ELISA kits (MLBIO, Shanghai, China), according to the manufacturers' instructions.

Proteomic analysis

We selected 10 SP-AKI patients and 10 SP-non-AKI patients matched for baseline characteristics (including gender, age, and absence of prior comorbidities).

NanoLC-MS/MS analysis was used to detect differentially expressed proteins between the two groups.

RNA extraction and sequencing

Lung and kidney tissues from five SP-AKI and five control mice were snap-frozen in liquid nitrogen and stored at −80°C. Total RNA was extracted using TRIzol, and quality assessed by NanoDrop and Bioanalyzer 2100 (RIN >7.0). Poly(A) RNA was enriched from 1 μg total RNA, fragmented, reverse-transcribed using SuperScript™ II, and ligated to adapters. Libraries (insert size ~300 bp) were sequenced on an Illumina NovaSeq 6000 platform with 2×150 bp paired-end reads (LC-Bio, Hangzhou).

Functional enrichment analysis

R package (version 4.2.2) "ClusterProfiler" was used for functional annotation of module genes to comprehensively explore the functional relevance of these module genes. Gene Ontology (GO) and the Kyoto Encyclopedia of Genes and Genomes (KEGG) were employed to assess relevant functional categories. and pathways with *P* value less than 0.05 were considered significant categories.

Single-cell RNA sequencing (scRNA-seq) data analysis

We analyzed multiple publicly available scRNA-seq datasets from the GEO database. The GSE210622 dataset (3) included kidney tissues from 8 severe AKI (stage 2–3) patients who died of respiratory infections, including 4 with COVID-19, and non-AKI controls. BALF scRNA-seq data from WT and Padi2/Padi4 double-knockout mice with Pseudomonas aeruginosa–induced pneumonia or sham treatment were obtained from GSE274823 (4). Additional datasets included GSE268542 (5), comprising lung

tissues from COPD patients infected ex vivo with influenza virus and bacteria; GSE280922 (6), a bleomycin-induced mouse lung injury model; and GSE239835 (7), profiling mouse lungs 48 hours after COVID-19 infection. These datasets were used for integrative single-cell transcriptomic analyses.

Unsupervised clustering and visualization

Raw scRNA-seq count matrices were analyzed using the Seurat package (v4.1.1) in R (v4.1.3). Genes detected in fewer than three cells were excluded. Cells expressing <200 or >10,000 genes, with mitochondrial gene content >10%, or >10% erythrocyte transcripts were removed. Batch effects were corrected and datasets integrated using reciprocal principal component analysis (RPCA). Data were normalized by library size, scaled to 10,000 transcripts per cell, and log transformed. Highly variable genes were identified using FindVariableFeatures. Principal component analysis (PCA) was performed on scaled data, and selected components were used for graph-based clustering (resolution = 0.9) and visualization using Uniform Manifold Approximation and Projection (UMAP) or t-distributed stochastic neighbor embedding (t-SNE).

Marker gene identification and cell-type annotation

Following normalization with SCTransform and RPCA-based integration using Seurat, clustering and UMAP visualization were performed using the top 2,000 highly variable genes and the first 30 principal components. Marker genes were identified using FindAllMarkers with thresholds of FDR-adjusted $P < 0.01$ and log2 fold change ≥ 0.25, retaining only positively associated genes expressed in $\geq 10\%$ of cells. Cell

clusters were annotated based on canonical markers from the CellMarker database (http://117.50.127.228/CellMarker/) (8) and published human kidney single-cell studies (9).

Renal tubular injury scoring

Renal tissues were fixed with 4% paraformaldehyde, embedded in paraffin, and sectioned at 4 μm. Sections were stained with hematoxylin and eosin (H&E) or periodic acid-Schiff (PAS) reagent. Tubular injury was independently assessed by two blinded observers using a semiquantitative scoring system based on the following criteria (10, 11): (1) tubular dilatation, (2) epithelial cell detachment or flattening, (3) cast formation, and (4) interstitial inflammation. Each criterion was scored on a scale of 0–4 (0: no injury; 1: < 5%; 2: 5%–25%; 3: 25%–75%; 4: > 75% tubular injury). Five random fields per section were scored, and the average score was calculated as the tubular injury index.

Lung injury scoring

Lung tissues were inflated with 4% paraformaldehyde, paraffin-embedded, and sectioned. H&E-stained sections were evaluated by two blinded observers using a semiquantitative scoring system. The acute lung injury score was adapted from the American Thoracic Society guidelines (12) and included: (1) alveolar wall thickening, (2) intra-alveolar hemorrhage, (3) neutrophil infiltration, and (4) hyaline membrane formation. Each parameter was scored 0-4 (0: absent; 1: mild; 2: moderate; 3: severe; 4: very severe). Five randomly selected non-overlapping fields per sample were assessed and averaged.

Extraction of neutrophils from mouse lung tissue

Lung tissues from 3 mice were minced and digested with collagenase type I. The digested suspension was filtered through a 70 μm nylon mesh to remove undigested fragments (13). Neutrophils were then isolated by Percoll density gradient centrifugation.

Neutrophil depletion and adoptive transfer

Mice were intraperitoneally injected with 250 μg of anti–mouse Ly6G monoclonal antibody (clone 1A8; Bio X Cell, BE0075-1) to deplete neutrophils, while control mice received an equal dose of rat IgG (ANC 284-810, Adipogen). After 24 hours, neutrophils were isolated from mouse kidney tissue by flow cytometric sorting and resuspended in 200 μL PBS. A total of 5×10^5 neutrophils in 10ul (14) were administered via subcapsular renal injection at the indicated time points for adoptive transfer or induction of the SP-AKI model. All mice used in adoptive transfer experiments were pre-treated with neutrophil depletion (15, 16).

Extraction of neutrophils from human whole blood

Peripheral venous blood (5 mL) was collected from healthy volunteers into anticoagulant tubes. Neutrophils were isolated via density gradient centrifugation, washed with cell washing solution, and residual red blood cells were lysed with red blood cell lysis buffer (17). Neutrophil purity was confirmed to be ≥ 95%.

The induced generation of NETs

NETs formation was induced by stimulating freshly isolated neutrophils with serum from AKI patients with negative blood cultures. NETs were collected after 3 hours of

stimulation(18).

The purification of NETs

The adherent layer of NETs and neutrophils was collected and centrifuged at 450 g for 10 min at 4 °C. The resulting cell-free, NETs-rich supernatant was then centrifuged at 18,000*g* for 10 min at 4°C. The supernatant was discarded, and the NETs-containing pellet was resuspended in cold PBS for subsequent experiments (19).

Co-culture of NETs with HK2 cells

Intervene HK2 cells with the above purified NETs and culture them in a 37°C constant-temperature incubator with 5% CO2 for 24 hours.

Immunofluorescence

Cells were cultured on glass slides or confocal dishes, fixed with 4% paraformaldehyde, permeabilized with 0.1% Triton X-100, and blocked with 10% goat serum. Primary antibodies against MPO and IL-33 (1:200) were incubated overnight at 4 °C, followed by fluorescent secondary antibodies. Nuclei were stained with DAPI, and images were captured by confocal microscopy. The primary antibodies used in this study are listed in **Supplemental Table 6**.

Flow cytometry identification of IL-33 and MPO in neutrophils

Single-cell suspensions from mouse blood, lung, and kidney were analyzed by flow cytometry to identify immune populations and assess IL-33 expression. Mouse neutrophils were defined as $CD11b^{+}Ly6G^{+}$ cells, and IL-33–positive populations were quantified. In human samples, neutrophils were identified as $CD66b^{+}$ cells, and IL-33 and MPO expression were analyzed. Data were acquired on a BD FACSCanto II

cytometer and analyzed using FlowJo software. Antibody details are listed in **Supplemental Table 7.**

Flow cytometric detection of apoptosis

After stimulation, cells were centrifuged at 250 *g* for 5 min at 4 °C. The supernatant was discarded and cells were resuspended in 500 μL PBS. Annexin V-FITC/PI (LiankeBio, Cat. No. AP101-60-kit) was added for double staining in the dark, followed by gentle mixing and incubation at 4 °C for 30 min. Apoptosis was then assessed by flow cytometry.

Statistical analysis

Multivariate analysis was conducted using stepwise backward logistic regression to identify independent risk factors for SP-AKI. The predictive performance of biomarkers was evaluated by calculating the area under the receiver operating characteristic curve (AUC). Correlations were assessed using Pearson's correlation coefficient (parametric) or Spearman's rank correlation coefficient (non-parametric), as appropriate. *P* value <0.05 was considered statistically significant.

Reference：

1. Kellum JA, Lameire N, and Group KAGW. Diagnosis, evaluation, and management of acute kidney injury: a KDIGO summary (Part 1). *Crit Care.* 2013;17(1):204-18.
2. Qu JM, and Cao B. Guidelines for the diagnosis and treatment of adult community acquired pneumonia in China (2016 Edition). *Zhonghua jie he he hu xi za zhi = Zhonghua jiehe he huxi zazhi = Chinese journal of tuberculosis*

and respiratory diseases. 2016;39(4):241-2.

3. Hinze C, Kocks C, Leiz J, Karaiskos N, Boltengagen A, Cao S, et al. Single-cell transcriptomics reveals common epithelial response patterns in human acute kidney injury. *Genome Med.* 2022;14(1):103.

4. Yu X, Song Y, Dong T, Ouyang W, Shao L, Quan C, et al. Loss of PADI2 and PADI4 ameliorates sepsis-induced acute lung injury by suppressing NLRP3+ macrophages. *JCI Insight.* 2024;9(22):e181686.

5. Sohail A, Waqas FH, Braubach P, Czichon L, Samir M, Iqbal A, et al. Differential transcriptomic host responses in the early phase of viral and bacterial infections in human lung tissue explants ex vivo. *Respir Res.* 2024;25(1):369.

6. Farooq H, Luehmann HP, Koenitzer JR, Heo GS, Sultan DH, Kulkarni DH, et al. Molecular imaging in experimental pulmonary fibrosis reveals that nintedanib unexpectedly modulates CCR2 immune cell infiltration. *EBioMedicine.* 2024;110:105431.

7. Khatun MS, Remcho TP, Qin X, and Kolls JK. Cell-intrinsic and -extrinsic effects of SARS-CoV-2 RNA on pathogenesis: single-cell meta-analysis. *mSphere.* 2023;8(5):e0037523.

8. Zhang X, Lan Y, Xu J, Quan F, Zhao E, Deng C, et al. CellMarker: a manually curated resource of cell markers in human and mouse. *Nucleic Acids Res.* 2019;47(D1):D721-D8.

9. Lake BB, Chen S, Hoshi M, Plongthongkum N, Salamon D, Knoten A, et al. A

single-nucleus RNA-sequencing pipeline to decipher the molecular anatomy and pathophysiology of human kidneys. *Nat Commun.* 2019;10(1):2832.

10. Furuichi K, Wada T, Iwata Y, Sakai N, Yoshimoto K, Kobayashi Ki K, et al. Administration of FR167653, a new anti-inflammatory compound, prevents renal ischaemia/reperfusion injury in mice. *Nephrol Dial Transplant.* 2002;17(3):399-407.
11. Jiang Z, Zhang J, and Lu Y. Protective Effects and Mechanisms of Rosuvastatin on Acute Kidney Injury Induced by Contrast Media in Rats. *Int J Nephrol.* 2020;2020:3490641.
12. Matute-Bello G, Downey G, Moore BB, Groshong SD, Matthay MA, Slutsky AS, et al. An official American Thoracic Society workshop report: features and measurements of experimental acute lung injury in animals. *Am J Respir Cell Mol Biol.* 2011;44(5):725-38.
13. Benjamin JT, Plosa EJ, Sucre JM, van der Meer R, Dave S, Gutor S, et al. Neutrophilic inflammation during lung development disrupts elastin assembly and predisposes adult mice to COPD. *J Clin Invest.* 2021;131(1):e139481.
14. Deng B, Ma M, Deng W, Zhang H, Du X, Pan B, et al. Single-cell RNA sequencing reveals the protective role of renal Cx3cr1(+) macrophages in cisplatin-induced acute kidney injury. *FEBS J.* 2025.
15. Pollenus E, Malengier-Devlies B, Vandermosten L, Pham TT, Mitera T, Possemiers H, et al. Limitations of neutrophil depletion by anti-Ly6G antibodies in two heterogenic immunological models. *Immunol Lett.*

2019;212:30-6.

16. Linde IL, Prestwood TR, Qiu J, Pilarowski G, Linde MH, Zhang X, et al. Neutrophil-activating therapy for the treatment of cancer. *Cancer Cell.* 2023;41(2):356-72 e10.

17. Hsu AY, Peng Z, Luo H, and Loison F. Isolation of Human Neutrophils from Whole Blood and Buffy Coats. *J Vis Exp.* 2021(175).

18. Lelliott PM, Momota M, Lee MSJ, Kuroda E, Iijima N, Ishii KJ, et al. Rapid Quantification of NETs In Vitro and in Whole Blood Samples by Imaging Flow Cytometry. *Cytometry A.* 2019;95(5):565-78.

19. Najmeh S, Cools-Lartigue J, Giannias B, Spicer J, and Ferri LE. Simplified Human Neutrophil Extracellular Traps (NETs) Isolation and Handling. *J Vis Exp.* 2015(98):52687.

Supplemental Figure 1. Proteomics-identified signaling pathways associated with SP-AKI in patient serum.

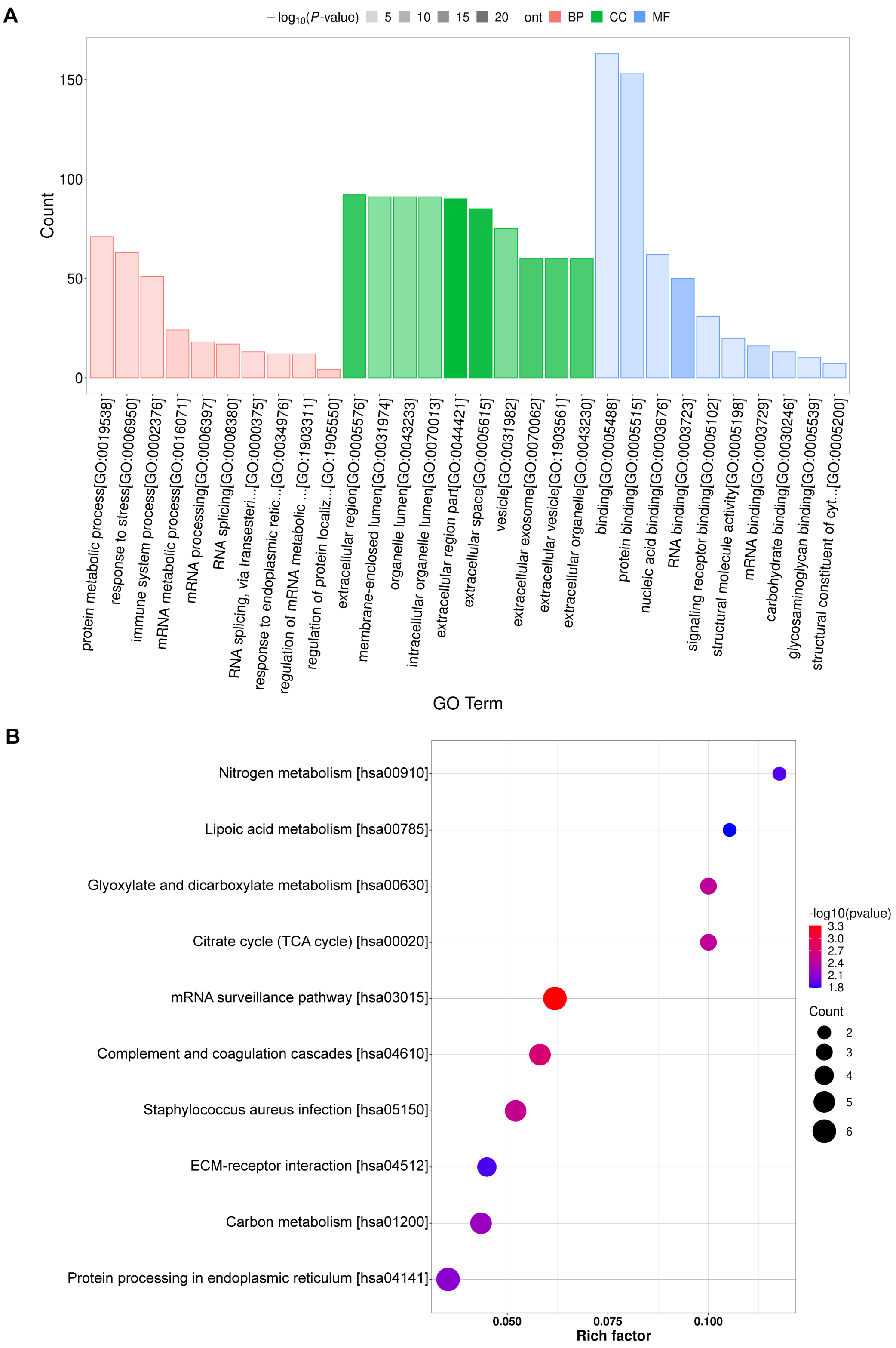

(A) Gene Ontology (GO) enrichment analysis of differentially expressed proteins. The vertical axis is the number of mapped differentially expressed proteins. Red indicates biological process annotation information, green indicates cell component annotation

information, blue indicates molecular function annotation information, and transparency indicates the size of *P* value. The darker the color, the smaller the *P* value. **(B)** Kyoto Encyclopedia of Genes and Genomes (KEGG) Enrichment Analysis Scatter Plot. The horizontal axis is the enrichment degree, and the vertical axis is the KEGG Pathway information. The size of the circle represents the number of differentially expressed proteins in the mapping pathway. The larger the circle, the greater the number; the color of the circle represents the *P* value size. The redder the color, the smaller the *P* value.

Supplemental Figure 2. Public scRNA-seq analyses reveal IL33+neutrophil enrichment and activation of NETs-related pathways in different lung injury.

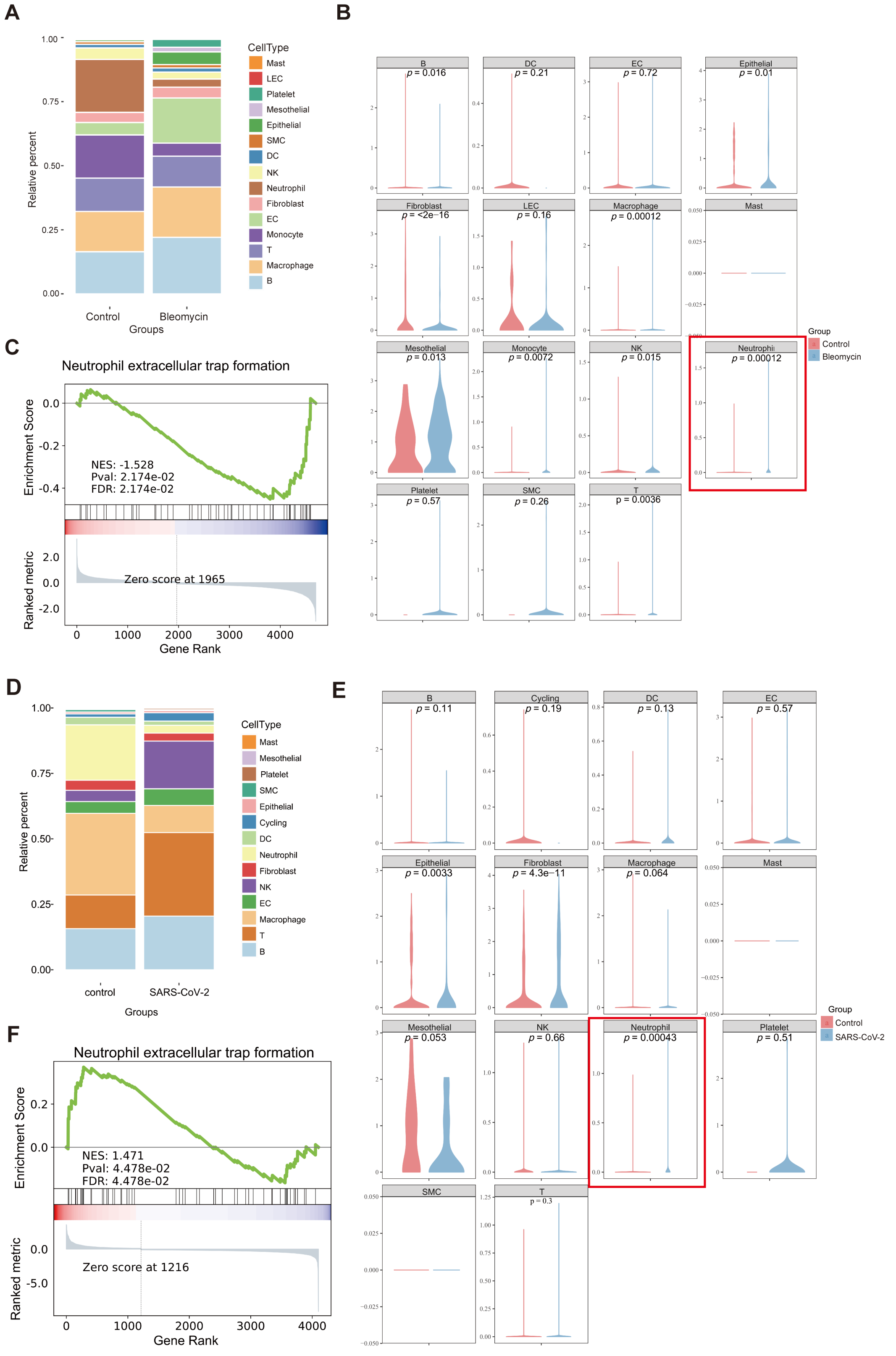

(A-C) Analysis of lung tissue from mice with bleomycin-induced lung injury

(GSE280922). (A) Relative proportions of major lung cell populations identified by single-cell RNA sequencing in control (CTL) and bleomycin-treated mice. Cell types are indicated by distinct colors. **(B)** Violin plots showing relative expression levels of IL33 genes across major lung cell populations in CTL and bleomycin-treated mice. P values indicate differences between groups for each cell type. **(C)** Gene set enrichment analysis (GSEA) showing enrichment of the NET formation pathway in lung tissues from bleomycin-treated mice compared with CTL mice. Normalized enrichment score (NES), nominal *P* value, and false discovery rate (FDR) are shown. **(D–F) Analysis of lung tissue from mice with SARS-CoV-2–induced lung injury (GSE239835). (D)** Relative proportions of major lung cell populations identified by single-cell RNA sequencing in CTL and SARS-CoV-2–infected mice. Cell types are indicated by distinct colors. **(E)** Violin plots showing relative expression levels of IL33 genes across major lung cell populations in CTL and SARS-CoV-2–infected mice. *P* values indicate differences between groups for each cell type. **(F)** GSEA showing enrichment of the NET formation pathway in lung tissues from SARS-CoV-2–infected mice compared with CTL mice. NES, nominal *P* value, and FDR are shown.

Supplemental Figure 3. Flowchart of patient enrollment in the severe pneumonia–acute kidney injury (SP-AKI) cohort.

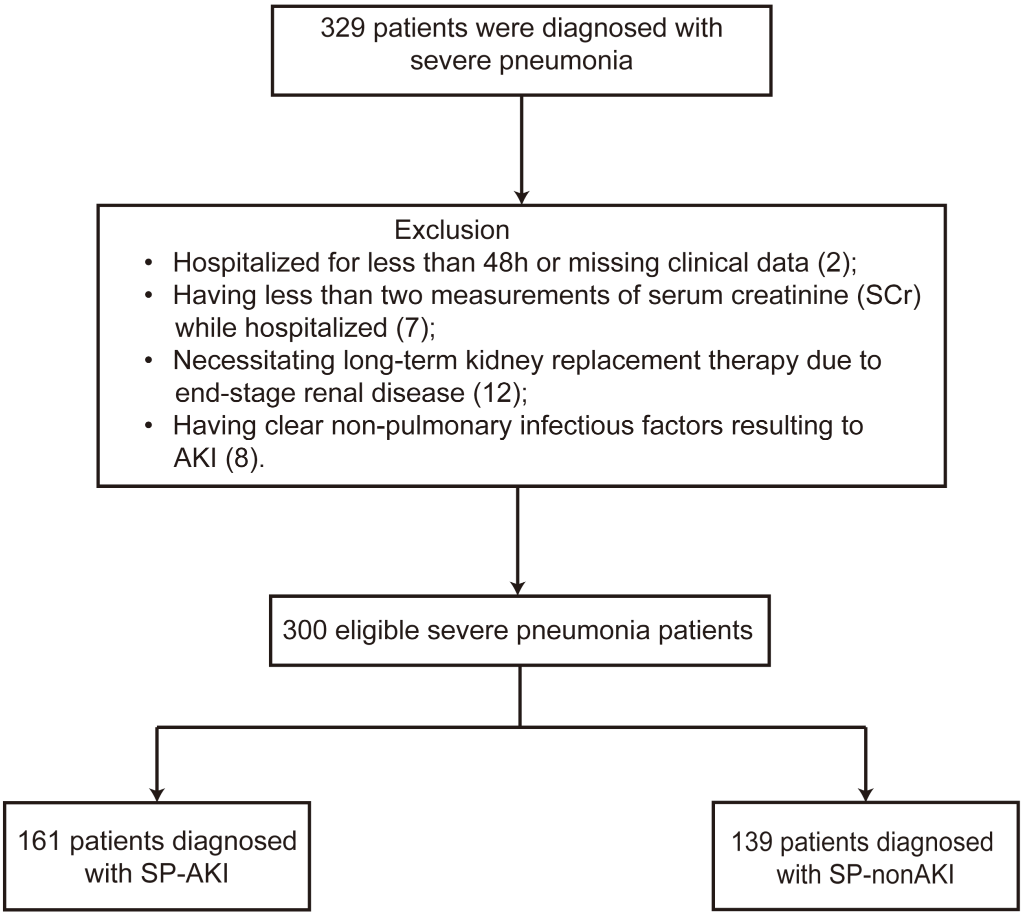

Supplemental Figure 4. Discriminative ability of circulating biomarkers for SP-AKI.

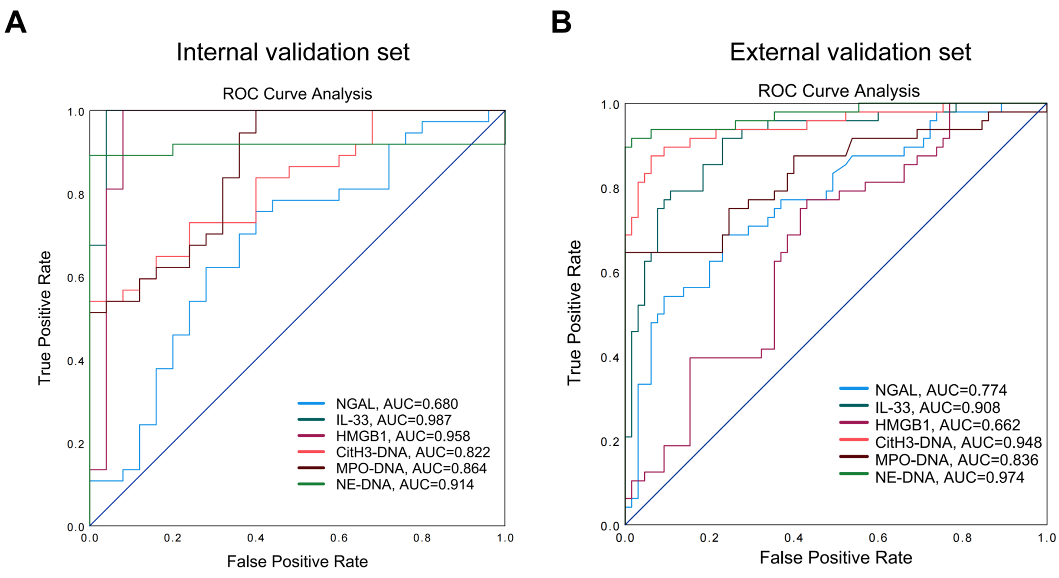

(A) Receiver operating characteristic (ROC) curve analysis of circulating biomarkers in the internal validation cohort. Biomarkers included neutrophil gelatinase-associated lipocalin (NGAL), interleukin-33 (IL-33), high-mobility group box 1 (HMGB1), citrullinated histone H3 (CitH3)–DNA complexes, myeloperoxidase (MPO)–DNA complexes, and neutrophil elastase (NE)–DNA complexes. **(B)** ROC curve analysis of the same biomarkers in the external validation cohort. The area under the ROC curve (AUC) was calculated to assess and compare the predictive performance of each biomarker for SP-AKI.

Supplemental Figure 5. RNA sequencing analysis of lung tissues in SP-AKI mouse model.

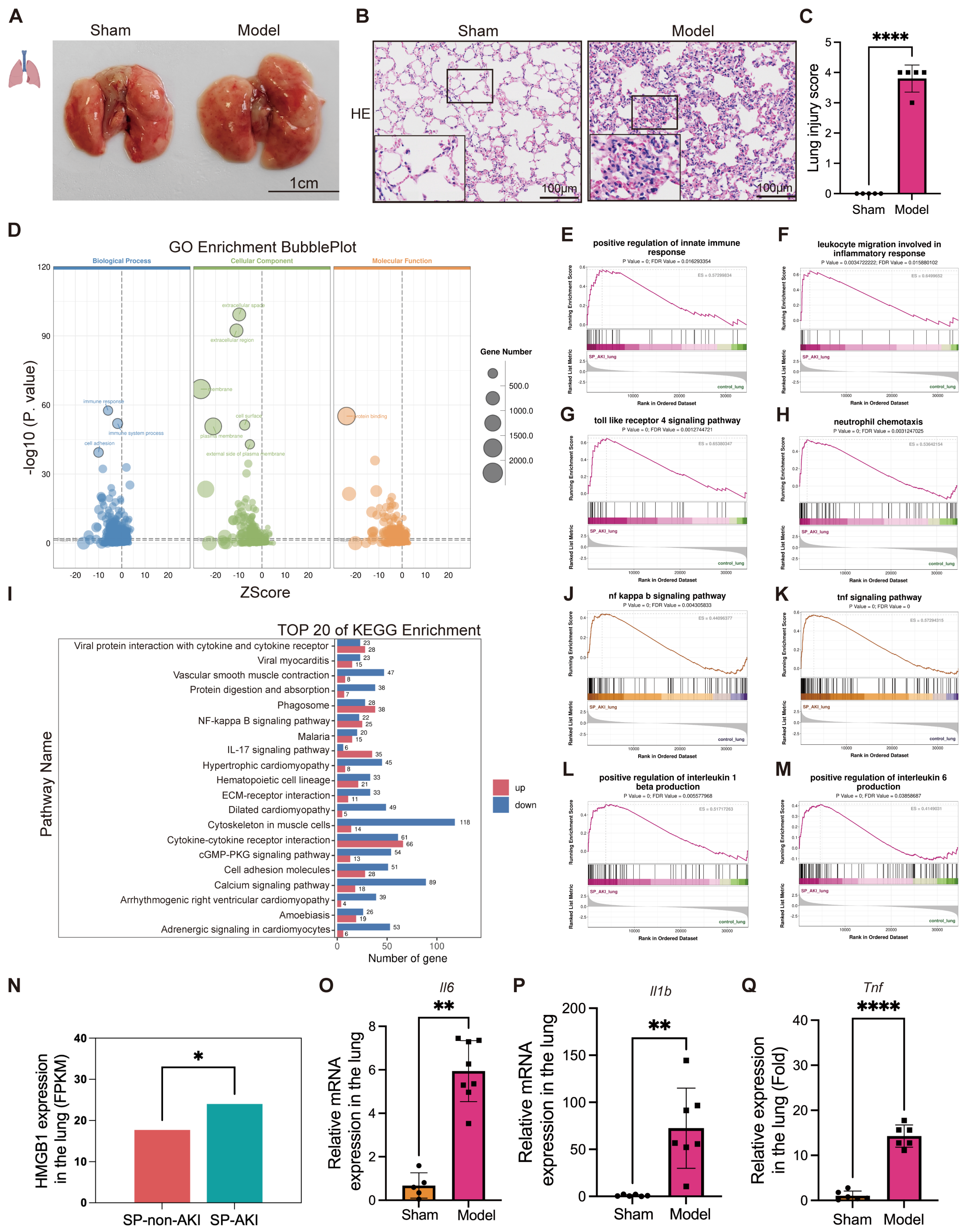

(A) Macroscopic images of mouse lung tissues in sham and model groups. Unit= cm. **(B)** Representative H&E-stained lung sections from sham and SP-AKI mice. Bar: 100μm. **(C)** Lung injury score according to Smith scoring method in two groups. **(D)** Gene Ontology (GO) enrichment analysis plot of differentially expressed genes in mouse lung tissue. Bubble size corresponds to the number of genes associated with each term, ranging from 500 to 2000. **(E-H)** GSEA enrichment plots for key innate immunity- and inflammation- related pathways in lung tissues of sham *vs.* model mice:

(E) Positive regulation of innate immune response; (F) Leukocyte migration involved in inflammatory response; (G) Toll - like receptor 4 signaling pathway; (H) Neutrophil chemotaxis. **(I)** Top 20 Kyoto Encyclopedia of Genes and Genomes (KEGG) pathway enrichment analysis in mouse lung tissue. Color indicates upregulated (red) or downregulated (blue) pathways. **(J-M)** GSEA enrichment plots for inflammatory signaling pathways in lung tissues of sham *vs.* model mice: (J) NF-κB signaling pathway; (K) TNF signaling pathway; (L) positive regulation of IL-1β production; (M) positive regulation of IL-6 production. **(N)** HMGB1 expression in lung tissues from SP-AKI and SP-non-AKI mouse. **(O-Q)** qPCR showed the expression of inflammatory factors in lung tissues, including IL-6, IL-1β and TNF-α. Data are presented as mean ± SD. n = 5 per group. *P* values were calculated by two-tailed t test, $^{**}P<0.01$, $^{****}P<0.0001$.

.

Supplemental Figure 6. RNA sequencing analysis of kidney tissues in SP-AKI mouse model.

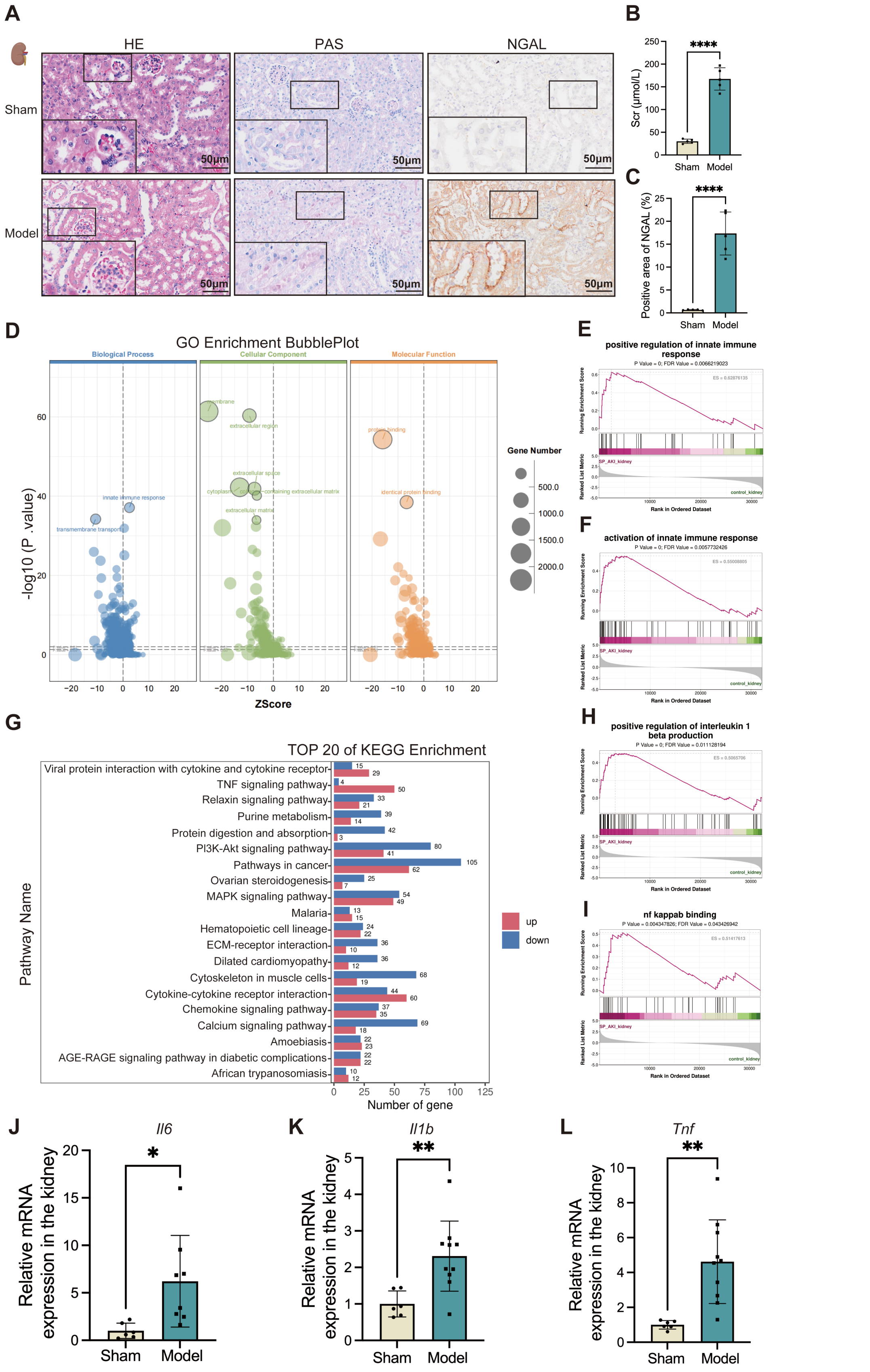

(A) Representative hematoxylin and eosin (H&E) staining, periodic acid–Schiff (PAS) staining, and NGAL immunohistochemistry (IHC) of kidney sections from sham and model mice. Scale bar: 50 µm. **(B)** Serum creatinine levels in sham and model groups. **(C)** Semi-quantitative analysis of the IHC results of NGAL in the kidneys of the two groups of mice. **(D)** Gene Ontology (GO) enrichment analysis plot of differentially expressed genes in mouse kidney tissue. Bubble size corresponds to the number of genes associated with each term, ranging from 500 to 2000. **(E-F)** GSEA enrichment plots for inflammatory signaling pathways in kidney tissues of sham *vs.* model mice: (E) Positive regulation of innate immune response; (F) Activation of innate immune response. **(G)** Top 20 Kyoto Encyclopedia of Genes and Genomes (KEGG) pathway enrichment analysis in mouse kidney tissue. Color indicates upregulated (red) or downregulated (blue) pathways. **(H-I)** GSEA enrichment plots for inflammatory signaling pathways in kidney tissues of sham *vs.* model mice: (H) Positive regulation of IL-1β production; (I) NFkappab binding. **(J-L)** qPCR showed the expression of inflammatory factors in mice kidney tissues, including IL-6 (J), IL-1β (K) and TNF-α (L). Data are presented as mean ± SD. n = 5 per group. *P* values were calculated by two-tailed t test, **P*<0.05, ***P*<0.01, *****P*<0.0001.

.

Supplemental Figure 7. In vivo half-life of adoptively transferred neutrophils in recipient mice and their temporal impact on kidney injury.

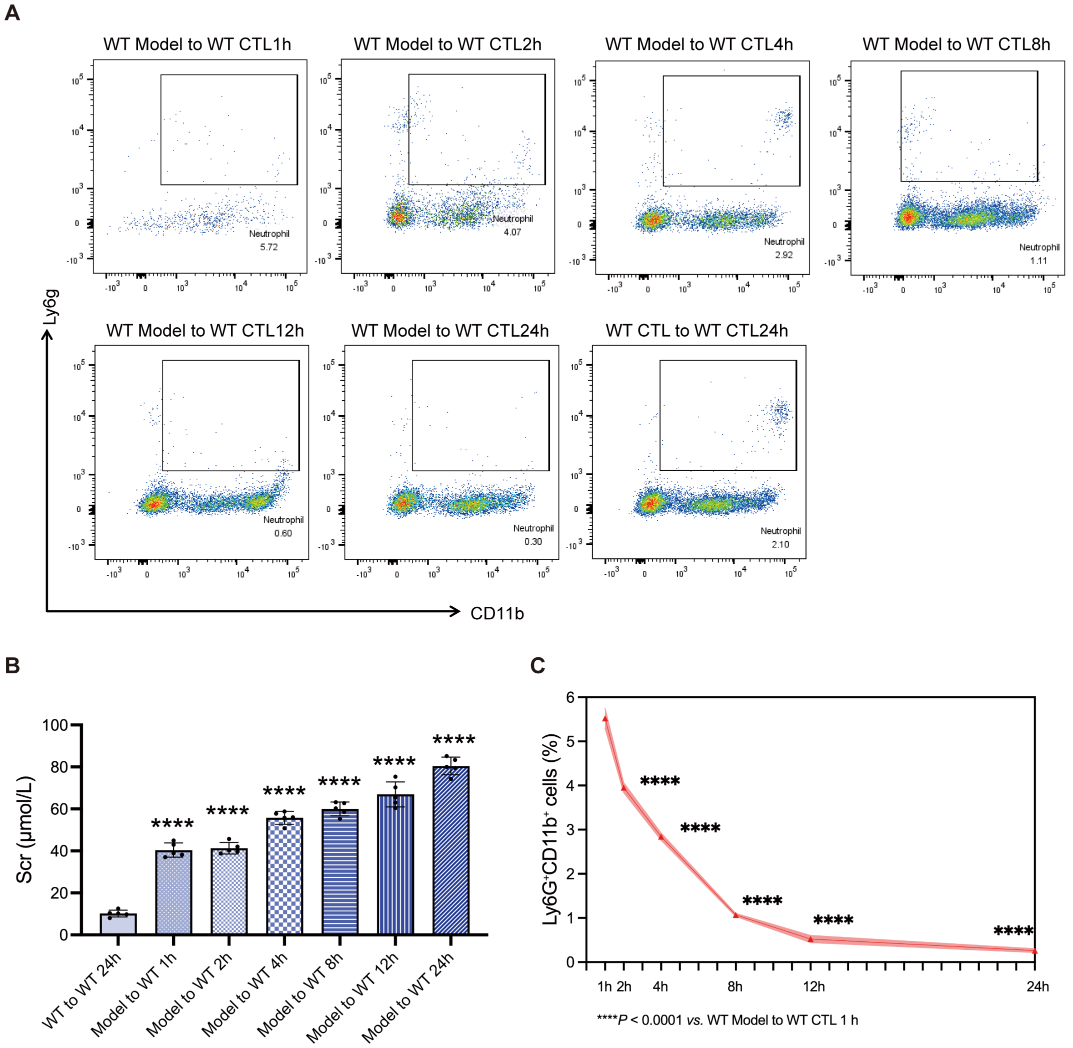

(A) Neutrophils (Ly6g$^+$CD11b$^+$) were isolated from the lung tissues of WT control and SP-AKI model mice by fluorescence-activated cell sorting (FACS) and subsequently adoptively transferred into WT control recipient mice. Recipient mice were pre-treated with intraperitoneal injection to deplete endogenous neutrophils prior to transfer. The number of Ly6g$^+$CD11b$^+$ neutrophils was monitored in recipients at 1, 2, 4, 8, 12, and 24 hours after transfer from WT model donors, alongside a 24-hour time point for recipients of WT control donor cells. **(B)** Bar graph showing serum creatinine levels in recipient mice under different adoptive transfer time. **(C)** Quantitative analysis of Ly6g$^+$CD11b$^+$ neutrophil counts (%) measured by flow cytometry in WT control recipient mice receiving cells from WT control donors at 1, 2, 4-, 8-, 12-, and 24-hours post-transfer. Data are presented as mean ± SD. n = 5 per group. *P* values were calculated by one-way ANOVA with post hoc Holm–Šídák test, *****P*<0.0001.

Supplemental Figure 8. IL-33 deficiency reduced HMGB1 release and NETs formation.

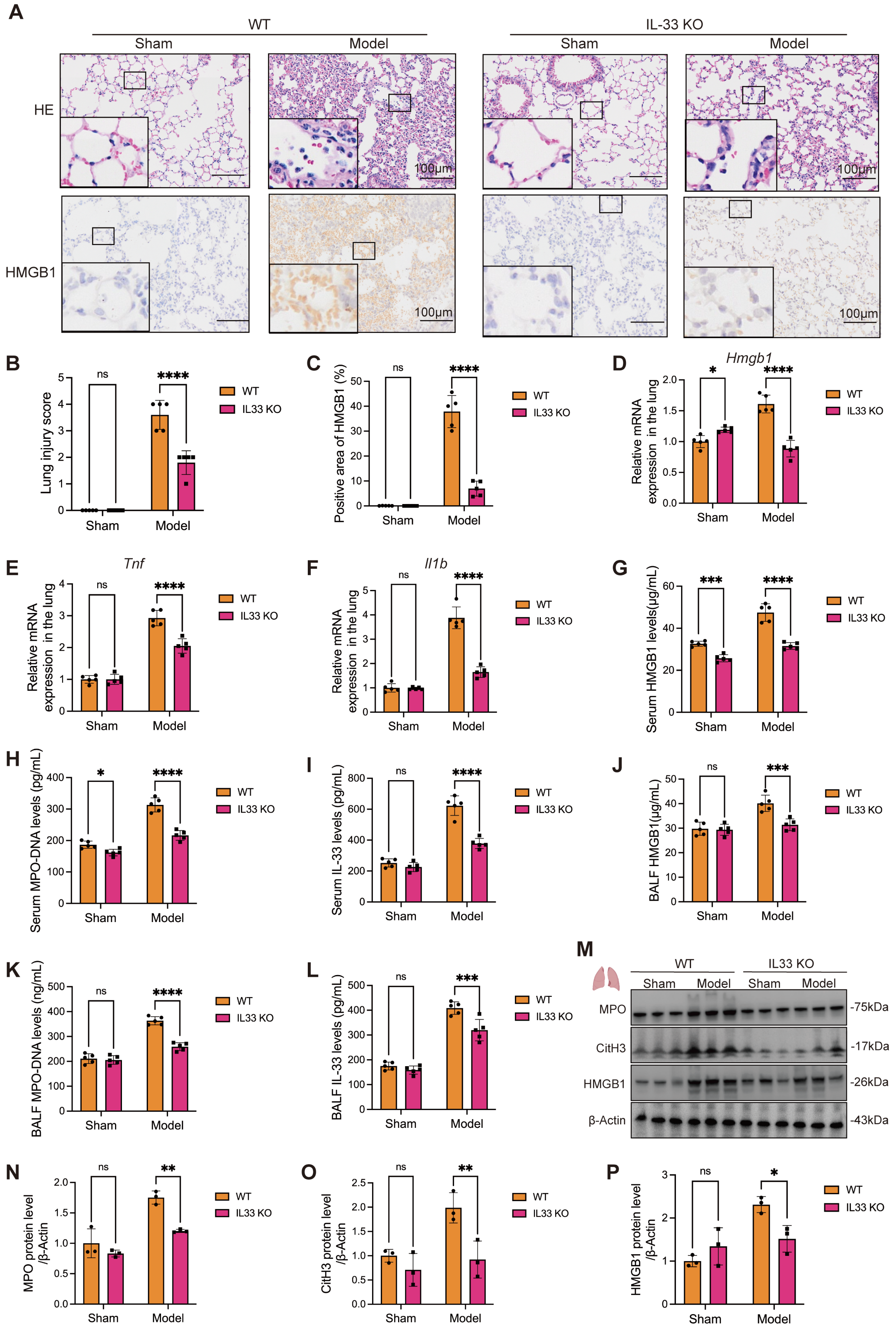

(A) Representative H&E staining and immunohistochemical staining of HMGB1 in lung sections from WT and IL-33 knockout (IL-33 KO) mice under sham and SP-AKI model conditions. Scale bar=100μm. **(B)** Lung injury score for WT and IL-33 KO

mice groups in figure A. **(C)** Quantification of HMGB1-positive areas in lung sections from the indicated groups. **(D-F)** Relative mRNA expression levels of HMGB1 (D), TNF-α (E), and IL-1β (F) in lung tissues from WT and IL-33 KO mice. **(G-I)** Serum levels of HMGB1 (G), MPO-DNA complexes (H), and IL-33 (I). **(J-L)** Levels of HMGB1 (J), MPO-DNA complexes (K), and IL-33 (L) in bronchoalveolar lavage fluid (BALF) from the indicated groups. **(I-L)** Expression of MPO and IL-33 in serum and BALF. **(M)** Western blot showed the expression of MPO, CitH3, and HMGB1 in the lungs of mice in each group, with β-actin as a loading control. **(N-P)** Semi-quantitative analysis of MPO (N), CitH3 (O), and HMGB1 (P) protein expression. Data are presented as mean ± SD. n = 5 per group. *P* values were calculated by two-way ANOVA with post hoc Holm–Šídák test, **P*<0.05, ***P*<0.01, ****P*<0.001, *****P*<0.0001, ns indicates no statistically significant difference.

Supplemental Figure 9. Therapeutic effects of targeting IL-33/HMGB1/NETs in SP-AKI.

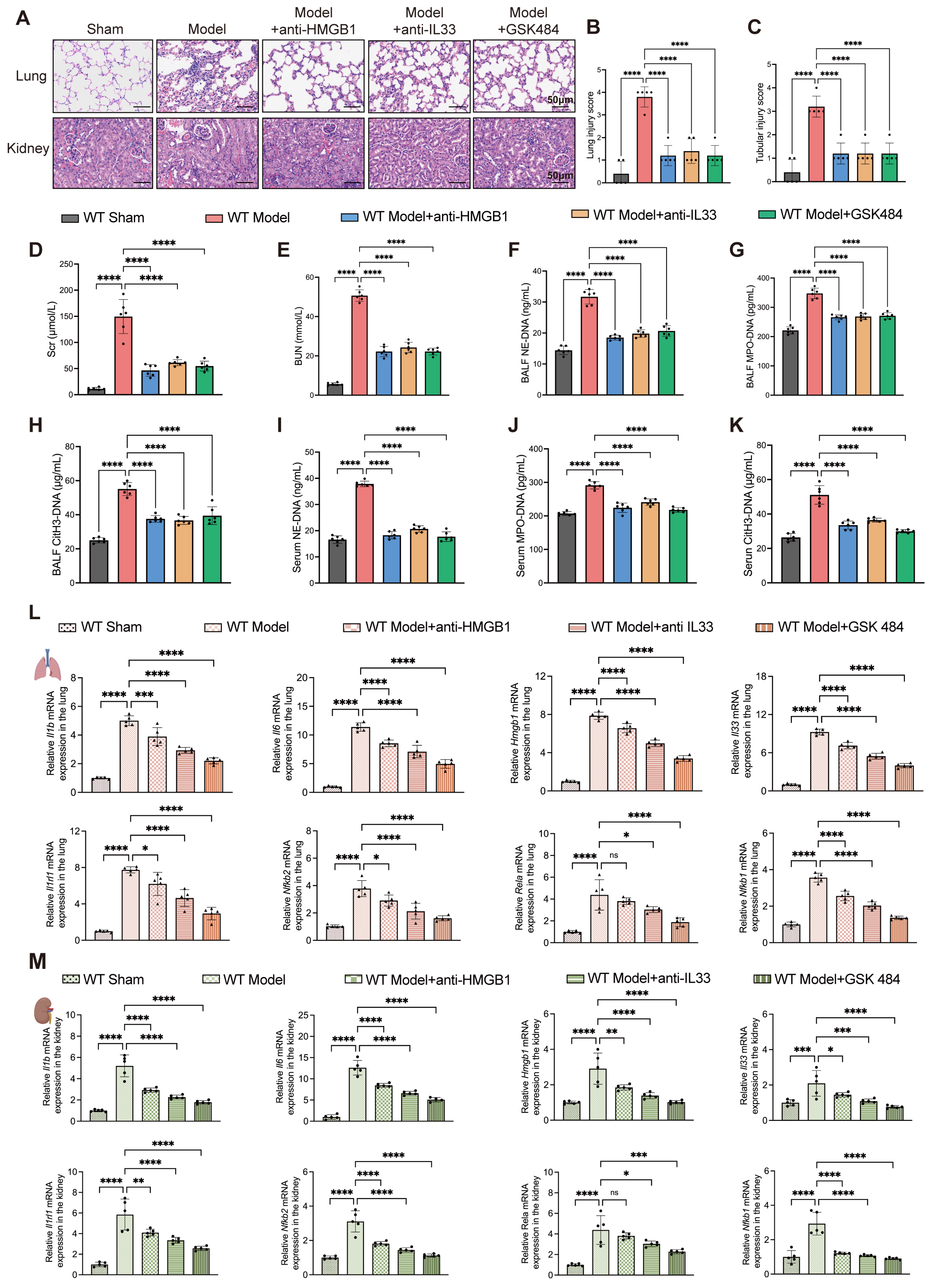

(A) Representative hematoxylin and eosin (H&E) staining of lung and kidney sections from the sham group, model group, model + anti-HMGB1 group (100ug/kg), model + anti-IL-33 group (60ug/kg), and model + GSK484 group (4mg/kg). Treatments were administered 6 hours after model induction. Scale bar: 50 μm. **(B-C)** The lung injury scores (B) and the renal tubular injury scores (C) for the mouse groups shown in

Figure A. **(D-E)** Serum creatinine and blood urea nitrogen levels in groups. **(F-K)** ELISA quantification of NE-DNA complexes (F, I), MPO-DNA complexes (G, J), and CitH3-DNA complexes (H, K) in serum and bronchoalveolar lavage fluid (BALF) from the mouse groups. **(L-M)** Relative mRNA expression levels of IL-1β, IL-6, HMGB1, IL-33, ST2, NFkB2, RelA and NFkB1 in lung (L) and kidney (M) tissues. Data are presented as mean ± SD. n = 5 per group. *P* values were calculated by one-way ANOVA with post hoc Holm–Šídák test, *$P<0.05$, **$P<0.01$, ***$P<0.001$, ****$P<0.0001$, ns indicates no statistically significant difference.

Supplemental Figure 10. Flow cytometric analysis of IL-33 expression in circulating immune cell subsets across experimental groups.

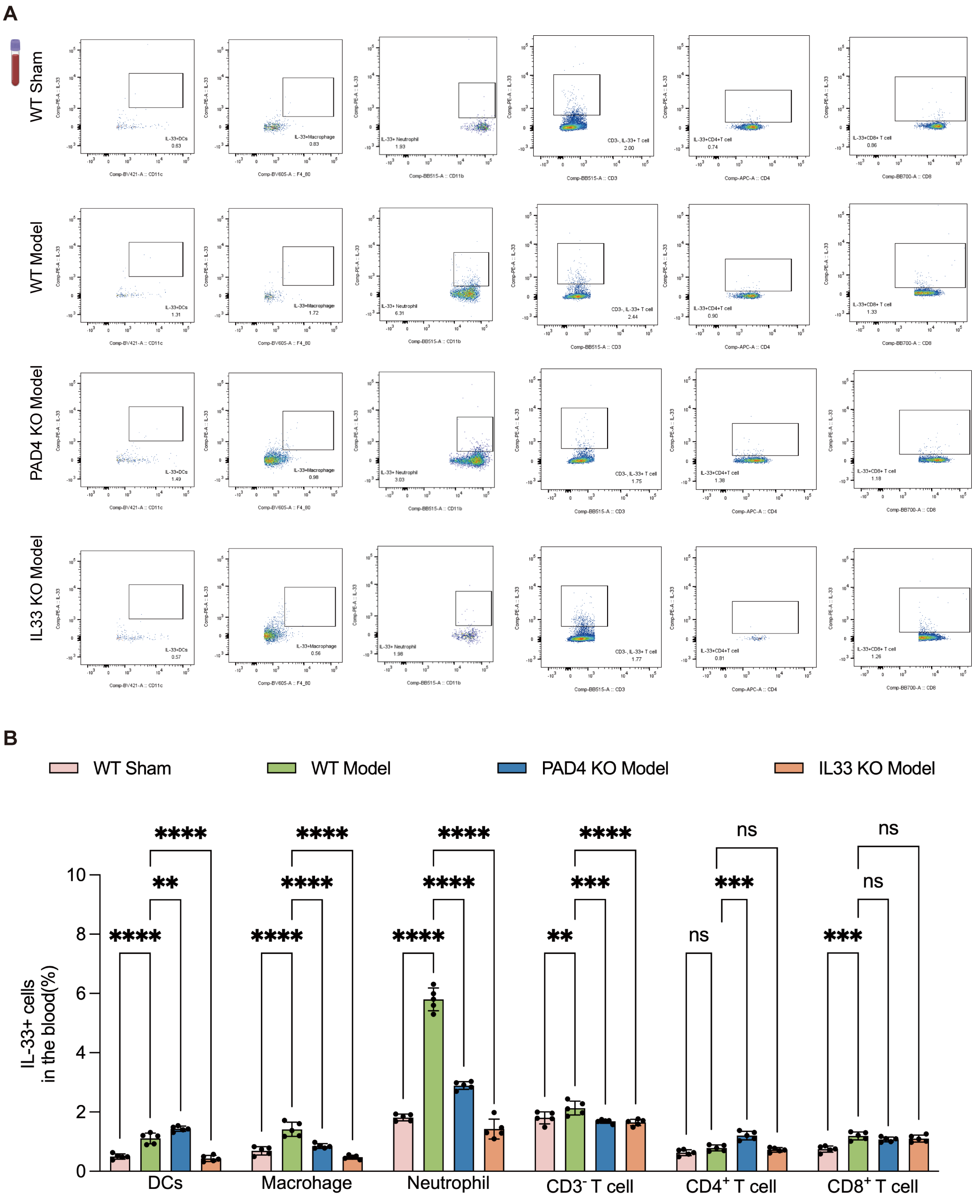

(A) IL-33 expression was analyzed by flow cytometry in circulating immune cell subsets—including dendritic cells, macrophages, neutrophils, $CD3^{-}$ T cells, $CD4^{+}$ T cells, and $CD8^{+}$ T cells—from four experimental groups: WT sham, WT model, PAD4 KO model, and IL-33 KO model. **(B)** Quantitative summary of the frequency of IL-33-positive cells within each immune subset. Data are presented as mean ± SD. n = 5 per group. *P* values were calculated by one-way ANOVA with post hoc Holm–Šídák test, $^{**}P<0.01$, $^{***}P<0.001$, $^{****}P<0.0001$, ns indicates no statistically significant difference.

Supplemental Figure 11. Flow cytometric analysis of IL-33 expression in kidney immune cell subsets across experimental groups.

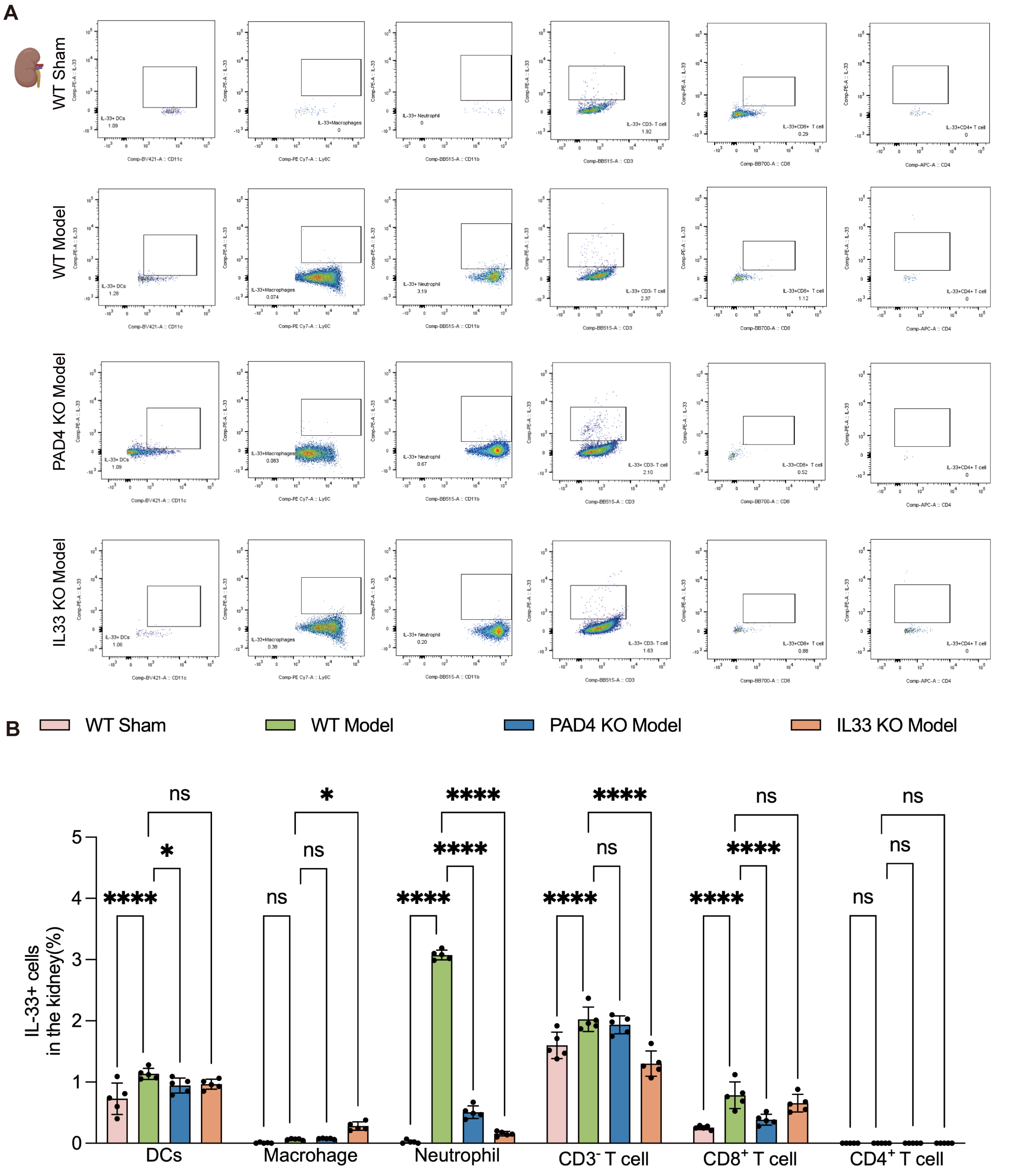

(A) IL-33 expression was analyzed by flow cytometry in kidney immune cell subsets—including dendritic cells, macrophages, neutrophils, $CD3^{-}$ T cells, $CD4^{+}$ T cells, and $CD8^{+}$ T cells—from four experimental groups: WT sham, WT model, PAD4 KO model, and IL-33 KO model. **(B)** Quantitative summary of the frequency of IL-33-positive cells within each immune subset. Data are presented as mean ± SD. n = 5 per group. *P* values were calculated by one-way ANOVA with post hoc Holm–Šídák test, **P*<0.05, ***P*<0.01, *****P*<0.0001, ns indicates no statistically significant difference.

Supplemental Figure 12. Flow cytometric analysis of IL-33 expression in lung immune cell subsets across experimental groups.

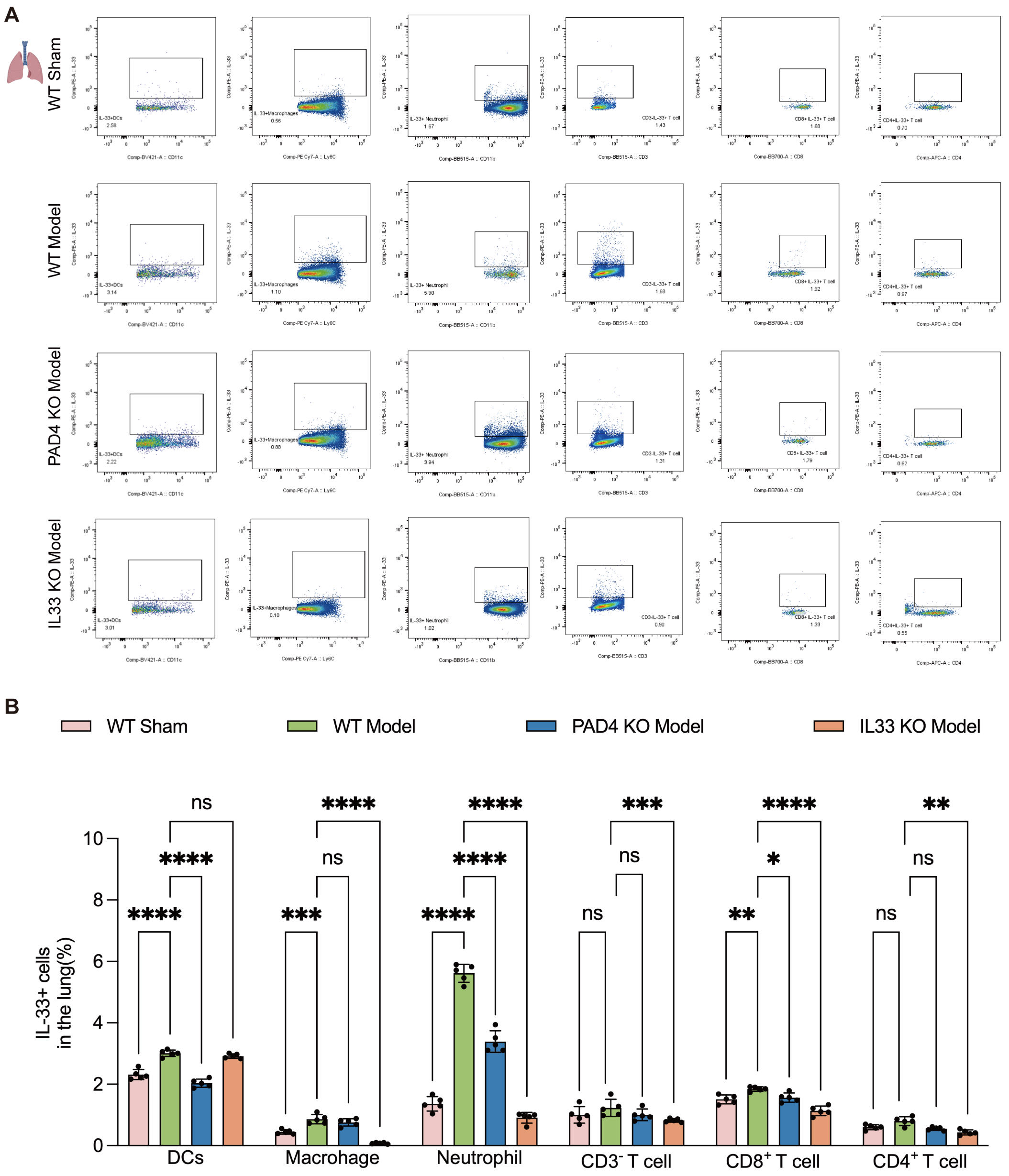

(A) IL-33 expression was analyzed by flow cytometry in lung immune cell subsets—including dendritic cells, macrophages, neutrophils, $CD3^{-}$ T cells, $CD4^{+}$ T cells, and $CD8^{+}$ T cells—from four experimental groups: WT sham, WT model, PAD4 KO model, and IL-33 KO model. Representative plots or gating strategies are shown. **(B)** Quantitative summary of the frequency of IL-33-positive cells within each immune subset. Data are presented as mean ± SD. n = 5 per group. *P* values were calculated by one-way ANOVA with post hoc Holm–Šídák test, $^{*}P<0.05$, $^{**}P<0.01$, $^{***}P<0.001$, $^{****}P<0.0001$, ns indicates no statistically significant difference.

Supplemental Table 1. Comparison of clinical data between SP-AKI and SP-non-AKI patients in severe pneumonia.

Characteristic	SP-AKI (n=161)	SP-non-AKI (n=139)	*P* value
**Clinical data**			
Male, n (%)	95 (59.0)	88 (63.3)	0.446
Age (yrs)	76 (67,85)	71 (59,82)	0.004
Admitted finger pulse oximetry (%)	95 (90,98)	96 (93,98)	0.131
Admission to the temperature (℃)	36.7 (36.5,37.0)	36.6 (36.3,36.8)	0.003
Admitted systolic blood pressure (mmHg)	126±23	131±19	0.056
Admitted diastolic blood pressure (mmHg)	71 (62,80)	75 (69,81)	0.012
Respiratory rate at admission (/min)	20 (18,22)	20 (18,21)	0.793
Heart rate at admission (/min)	89 (78,106)	86 (77,97)	0.113
APACHE II	17 (12,22)	11 (8,13)	<0.001
SOFA	6 (4,7)	3 (2,5)	<0.001
CURB-65	2 (1,3)	1 (1,2)	<0.001
Chronic kidney disease, n (%)	19 (11.8)	4 (2.9)	0.004
Chronic pulmonary disease, n (%)	4 (2.5)	10 (7.2)	0.054
Hypertension, n (%)	100 (62.1)	66 (47.5)	0.011
Diabetes, n (%)	52 (32.3)	36 (25.9)	0.225
**Laboratory indicators:**			
WBC (×10^9/L)	8.83 (5.71,13.86)	7.49 (5.14,9.60)	0.004
Neutrophils (×10^9/L)	7.08 (4.16,11.89)	5.41 (3.42,7.83)	0.002
Lymphocyte count (×10^9/L)	0.80 (0.55,1.39)	1.42 (0.68,5.20)	<0.001
Hemoglobin (g/L)	112 (95,128)	121 (99,132)	0.120
Platelet count (×10^9/L)	165 (125,228)	183 (133,265)	0.047
HCT (%)	34.3 (30.0,38.7)	37.0 (31.9,40.4)	0.017
PT (s)	12.80 (11.80,14.35)	12.30 (11.70,13.20)	0.003
APTT (s)	32.30 (29.25,36.30)	30.60 (28.60,32.75)	0.001
D-dimer (ug/ml)	0.78 (0.38,2.95)	1.50 (0.31,2.42)	0.082
PCT (ng/ml)	0.49 (0.13,2.38)	0.11 (0.07,0.43)	<0.001
Serum sodium	138.0±9.4	137.3±6.7	0.483

(mmol/L)			
Serum potassium (mmol/L)	4.12±0.85	3.77±0.56	<0.001
Serum albumin (g/L)	33.1±5.4	34.4±5.1	0.033
ALT (u/L)	23.3 (14.0,35.8)	18.6 (11.6,35.0)	0.290
AST (u/L)	31.0 (20.0,51.0)	24.0 (17.0,44.0)	0.008
Total bilirubin (μmol/L)	9.7 (6.4,14.7)	10.1 (7.0,14.9)	0.457
BUN (mmol/L)	10.90 (6.71,17.80)	6.00 (4.60,7.90)	<0.001
Scr at admission (μmol/L)	109.0 (69.0,193.8)	69.5 (56.0,86.9)	<0.001
Uric acid (μmol/L)	334 (225,481)	224 (175,298)	<0.001
Hematuria, n (%)	75 (53.2)	27 (32.1)	0.002
Proteinuria, n (%)	94 (58.4)	43 (30.9)	<0.001
Blood culture results			
Negative blood culture, n (%)	61 (37.9)	34 (23.5)	<0.001
Gram-positive cocci, n (%)	13 (8.1)	6 (4.3)	<0.001
Gram-negative bacilli, n (%)	19 (11.8)	2 (1.4)	<0.001
Fungi, n (%)	8 (5.0)	0 (0)	<0.001
COVID-19, n (%)	115 (71.4)	74 (53.2)	<0.001
**Outcome:**			
Death, n (%)	39 (24.2)	5 (3.6)	<0.001
CRRT, n (%)	31 (19.3)	0 (0.0)	<0.001
**Biomarkers:**			
NE-DNA (ng/ml)	20.32 (16.73,26.97)	13.53 (10.85,15.30)	<0.001
CitH3-DNA (ug/ml)	33.56 (27.76,40.89)	20.47 (16.84,23.61)	<0.001
MPO-DNA (pg/ml)	358.22 (246.74,517.10)	213.59 (172.68,238.98)	<0.001
IL-33 (pg/mL)	214.93 (154.80,285.51)	42.58 (22.21,168.45)	<0.001
HMGB1 (μg/mL)	33.09 (27.77,44.82)	10.78 (8.00,30.15)	<0.001
NGAL (ng/mL)	297.10 (238.27,372.67)	204.95 (165.38,274.06)	<0.001

The normality of continuous variables was rigorously evaluated using the Kolmogorov-Smirnov test. Parametric analyses (expressed as mean ± standard deviation) were applied to normally distributed data, while non-parametric (expressed as median with interquartile range) were employed for non-normally distributed variables. Categorical variables were compared via the chi - square test, with results reported as percentages. *P* values <0.05 indicates that the difference was statistically significant.

Abbriation: WBC, white blood cells; HCT, hematocrit; PT, prothrombin time; APTT, activated prothrombin time; PCT, procalcitonin; ALT, alanine aminotransferase; AST,

aspartate aminotransferase; BUN, blood urea nitrogen; CRRT, continuous renel replacement therapy; NE, neutrophil elastase; MPO, myeloperoxidase; CitH3, Citrullinated Histone H3; IL-33, interleukin-33; HMGB1, high mobility group box 1; NGAL, neutrophil gelatinase-associated lipocalin.

Supplemental Table 2. Multivariate logistic analysis of independent risk factors for SP-AKI.

Variables	Univariate OR (95% CI)	*P* value	Multivariable OR (95% CI)	*P* value
Age (yrs)	1.020（1.005-1.035）	0.009		
Admission to the temperature (℃)	1.421（1.005-2.008）	0.047		
APACHE II	1.162（1.101-1.226）	<0.001	1.077(1.014-1.145)	0.017
SOFA	1.387(1.219-1.579)	<0.001		
CURB-65	2.398(1.724-3.335)	<0.001		
Chronic kidney disease, n (%)	4.787（1.595-14.369）	0.005		
Hypertension, n (%)	1.813（1.144-2.874）	0.011		
Mechanical ventilation	4.414（2.620-7.437）	<0.001	2.374(1.080-5.219)	<0.001
WBC (×10^9/L)	1.084（1.035-1.136）	0.001		
Neutrophils (×10^9/L)	1.112（1.055-1.172）	<0.001	1.099(1.014-1.191)	0.021
Platelet count (×10^9/L)	0.997(0.994-1.000)	0.022	0.994(0.989-0.998)	0.007
HCT (%)	0.968(0.939-0.999)	0.041		
APTT (s)	1.055(1.015-1.098)	0.007		
D-dimer (ug/ml)	0.999(0.999-1.000)	0.019		
PCT (ng/ml)	1.145(1.034-1.267)	0.009		
Serum albumin (g/L)	0.954(0.913-0.996)	0.034		
BUN (mmol/L)	1.210(1.139-1.286)	<0.001		
Scr at admission (μmol/L)	1.015(1.010-1.020)	<0.001	1.016(1.008-1.024)	<0.001
Uric acid (μmol/L)	1.007（1.005-1.009）	<0.001		
Hematuria, n (%)	2.093（1.315-3.331）	0.002		
Proteinuria, n (%)	3.132（1.944-5.047）	<0.001	2.400(1.196-4.817)	0.014
Negative blood culture, n (%)	0.336（0.128-0.885）	0.027		
Gram-negative bacilli, n (%)	4.878（1.079-22.053）	0.039		

Variables with *P* values < 0.05 from univariate logistic regression were included in a multivariate logistic regression model to adjust for potential confounders. Data were presented as Odds ratios (ORs) and 95% confidence intervals (CIs). *P* values <0.05 indicates that the difference was statistically significant. The variables adjusted

include age, admitted finger pulse oximetry, admission to the temperature, admitted systolic blood pressure, heart rate at admission, COVID-19, chronic kidney disease, hypertension, WBC, HCT, APTT, D-dimer, PCT, serum potassium, serum albumin, AST, BUN, uric acid, hematuria. Sample size: The analysis was conducted on data from patients with severe pneumonia (n=300).

Supplemental Table 3. Expression levels of each biomarker in the three datasets.

Characteristic	Training set (n=125)			Internal validation set (n=62)			External validation set(n=113)		
	AKI (n=76)	Non-AKI (n=49)	*P* value	AKI (n=37)	Non-AKI (n=25)	*P* value	AKI (n=48)	Non-AKI (n=65)	*P* value
**Biomarkers:**									
NE-DNA (ng/ml)	20.56 (16.49, 27.39)	13.53 (11.93, 14.33)	<0.001	19.40 (16.51-23.29)	13.25 (12.34-14.34)	<0.001	31.54 (19.92, 39.27)	15.31 (9.88, 16.40)	<0.001
MPO-DNA (pg/ml)	278.56 (244.42, 506.70)	219.62 (174.77, 253.48)	<0.001	327.64 (247.92-503.02)	220.98 (179.13-239.44)	<0.001	436.29 (225.55, 542.24)	208.20 (165.09, 227.98)	<0.001
CitH3-DNA (ug/ml)	30.63 (21.42, 36.29)	18.56 (13.89, 24.42)		33.09 (27.75-38.50)	19.76 (15.92-23.39)		40.45 (31.59, 49.18)	21.02 (17.99, 23.79)	
NGAL (ng/mL)	321.26 (273.51, 379.32)	269.94 (217.74, 313.71)	0.017	341.58 (297.28-385.32)	270.51 (224.81-299.40)	<0.001	216.77 (177.51, 245.18)	167.67 (145.15, 189.37)	<0.001
IL-33 (pg/mL)	169.28 (145.66, 214.19)	24.65 (13.95, 32.94)	<0.001	170.55 (144.38-227.80)	22.39 (15.50-27.34)	<0.001	327.03 (280.86, 377.87)	171.45 (148.12, 247.75)	<0.001
HMGB1 (μg/mL)	30.01 (26.50, 36.01)	9.16 (7.07, 10.75)	<0.001	34.37 (27.73-43.51)	8.99 (7.75-10.52)	<0.001	33.66 (31.26, 50.88)	30.45 (22.83, 40.60)	0.003

Intergroup comparisons employed Student's t-test for continuous variables with normal distribution, data are expressed as mean ± standard deviation (SD). *P* values <0.05 indicates that the difference was statistically significant. For each biomarker in patients with severe pneumonia, the Benjamini/Hochberg (B/H) method was employed to perform multiple testing on two - sided *P* values. All *P* values were adjusted using the false discovery rate (FDR) correction, with adjusted *P* values <0.001.

Abbriation：NE, neutrophil elastase; MPO, myeloperoxidase; IL-33, interleukin-33; HMGB1, high mobility group box 1; NGAL, neutrophil

gelatinase-associated lipocalin.

Supplemental Table 4. Sensitivity and specificity of biomarkers at different cutoff values.

Biomarkers	Sensitivity	Specificity	Positive predictive value	Negative predictive value
NE-DNA (ng/ml)				
13.09	0.894	0.417	0.640	0.773
15.93	0.789	0.835	0.847	0.773
20.56	0.466	1.000	1.000	0.618
CitH3-DNA (ug/ml)				
19.74	0.919	0.446	0.658	0.827
25.45	0.801	0.849	0.860	0.787
34.69	0.466	1.000	1.000	0.618
MPO-DNA (pg/ml)				
213.59	0.963	0.496	0.689	0.920
246.09	0.758	0.791	0.808	0.738
389.76	0.466	1.000	1.000	0.618
NGAL (ng/mL)				
188.32	0.901	0.403	0.636	0.778
261.10	0.652	0.676	0.700	0.627
327.90	0.398	0.921	0.853	0.569
IL-33 (pg/mL)				
74.28	0.994	0.532	0.711	0.987
163.72	0.689	0.719	0.740	0.667
253.66	0.373	0.892	0.800	0.551
HMGB1 (μg/mL)				
11.30	1.000	0.540	0.716	1.000
28.65	0.696	0.727	0.747	0.673
36.75	0.335	0.849	0.720	0.524

The medians of 25%, 50% and 75% were used as cutoff values respectively. Abbriation: NE, neutrophil elastase; MPO, myeloperoxidase; IL-33, interleukin-33; HMGB1, high mobility group box 1; NGAL, neutrophil gelatinase-associated lipocalin.

Supplemental Table 5. Primer sequences used in this study.

Primer	Direction	Sequence
mouse IL-6	Forward	TAGTCCTTCCTACCCCAATTTCC
mouse IL-6	Reverse	TTGGTCCTTAGCCACTCCTTC
mouse IL-1β	Forward	GCAACTGTTCCTGAACTCAACT
mouse IL-1β	Reverse	ATCTTTTGGGGTCCGTCAACT
mouse TNFα	Forward	CCCTCACACTCAGATCATCTTCT
mouse TNFα	Reverse	GCTACGACGTGGGCTACAG
mouse IL33	Forward	CTACTGCATGAGACTCCGTTCTG
mouse IL33	Reverse	AGAATCCCGTGGATAGGCAGAG
mouse ST2	Forward	GGATTGAGGTTGCTCTGTTCTGG
mouse ST2	Reverse	TCGGGCAGAGTGTGGTGAACAA
mouse HMGB1	Forward	GGCGAGCATCCTGGCTTATC
mouse HMGB1	Reverse	GGCTGCTTGTCATCTGCTG
mouse P50/P105	Forward	GGAGGCATGTTCGGTAGTGG
mouse P50/P105	Reverse	CCCTGCGTTGGATTTCGTG
mouse P52/P100	Forward	GGCCGGAAGACCTATCCTACT
mouse P52/P100	Reverse	CTACAGACACAGCGCACACT
mouse RelA (P65)	Forward	AGGCTTCTGGGCCTTATGTG
mouse RelA (P65)	Reverse	TGCTTCTCTCGCCAGGAATAC
mouse GAPDH	Forward	AGGTCGGTGTGAACGGATTTG
mouse GAPDH	Reverse	TGTAGACCATGTAGTTGAGGTCA

Supplemental Table 6. Specific primary antibodies used in this study.

	Antigen	Species	Supplier	Cat #	Dilution
**Western blotting (WB)**	MPO	Rabbit	Abcam	ab208670	1:1000
	CitH3	Rabbit	Abcam	ab232939	1:1000
	HMGB1	Rabbit	Proteintech	10829-1-AP	1:1000
	IL-33	Mouse	Proteintech	66235-1-Ig	1:1000
	ST2	Rabbit	Proteintech	11920-1-AP	1:1000
	RelA	Mouse	Proteintech	66535-1-Ig	1:1000
	GAPDH	Mouse	Proteintech	60004-1-Ig	1:10000
	β-actin	Mouse	Proteintech	66009-1-Ig	1:10000
**Immunohistochemistry (IHC)**	HMGB1	Rabbit	Proteintech	10829-1-AP	1:200
	Ly6g	Rabbit	Aifang Biological	AFRM0008	1:200
	ST2	Rabbit	Proteintech	11920-1-AP	1:200
	NFκB	Rabbit	Cell Signaling Technology	#3033	1:200
	NGAL	Rabbit	Proteintech	26991-1-AP	1:200
**Immunofluorescence (IF)**	Ly6g	Rabbit	Aifang Biological	AFRM0008	1:200
	MPO	Rabbit	Abcam	ab208670	1:200
	PAD4	Rabbit	Proteintech	17373-1-AP	1:200
	IL-33	Mouse	Proteintech	66235-1-Ig	1:200
	NFkB	Rabbit	Proteintech	#3033	1:200

Supplemental Table 7. Antibodies used for flow cytometry.

	Antigen	Supplier	Cat #
**Human samples**	CD66b	BioLegend	305105
	IL-33	Abcam	ab275607
	MPO	BioLegend	78079
**Mouse samples**	CD45	BD Horizon	564279
	CD11b	BD Pharmingen	561688
	Ly6G	Invitrogen	46-9668-80
	IL-33	Invitrogen	MA5-23640
	CD11c	BD Horizon	565452
	F4/80	BioLegend	123133
	CD3	BioLegend	100204
	CD4	BioLegend	100516
	CD8	BD Horizon	574856
	MHC II	BD Horizon	570707
	Ly6C	BD Horizon	571412
