# Supplementary figures and images for: HMGB1-mediated formation of IL-33–abundant NETs drives lung-to-kidney injury in severe pneumonia–associated acute kidney injury
Source: JCI Insight. 2026 May 22;11(10):e191979. doi: 10.1172/jci.insight.191979 (PMC13232728; doi:10.1172/jci.insight.191979)

## Figure 2F

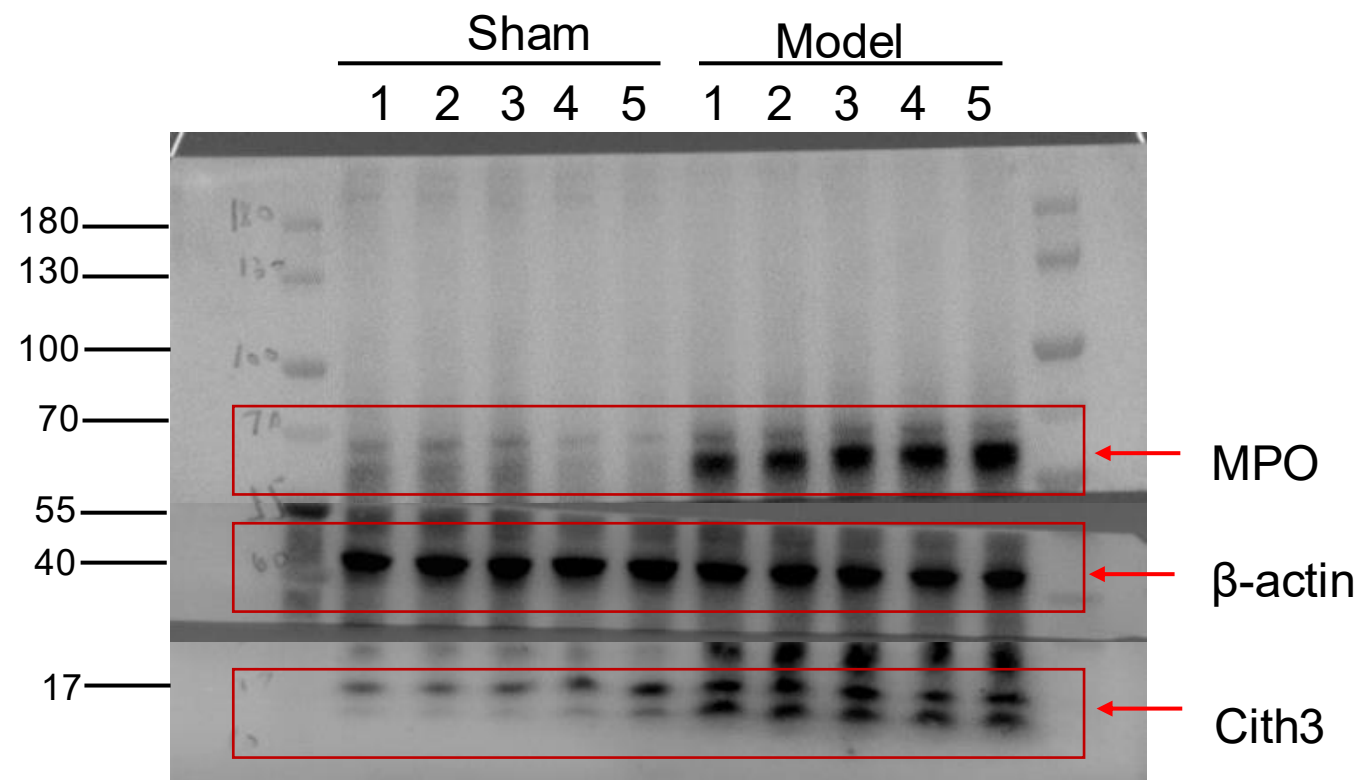

Figure 2G

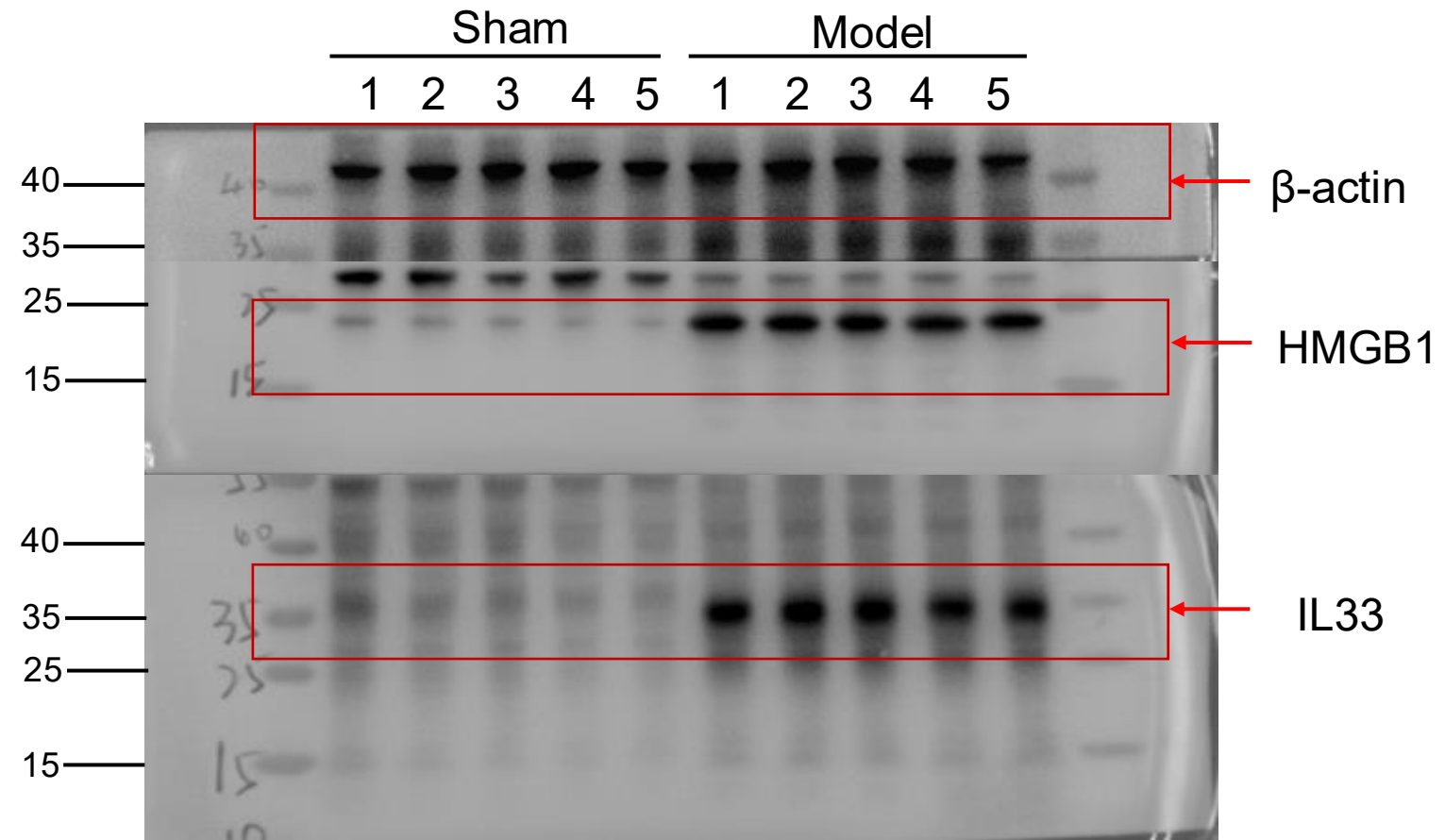

Figure 3G

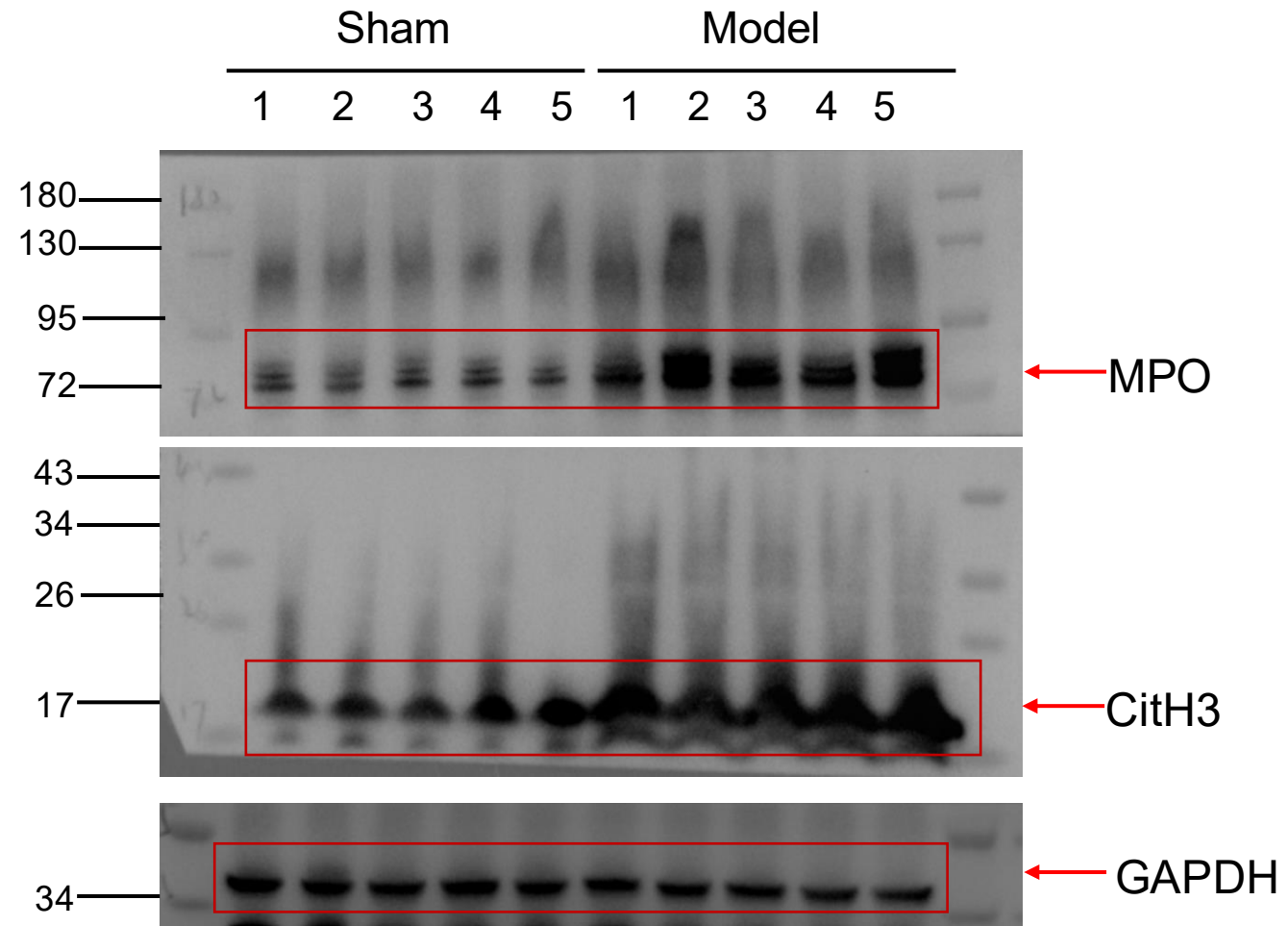

Figure 5A

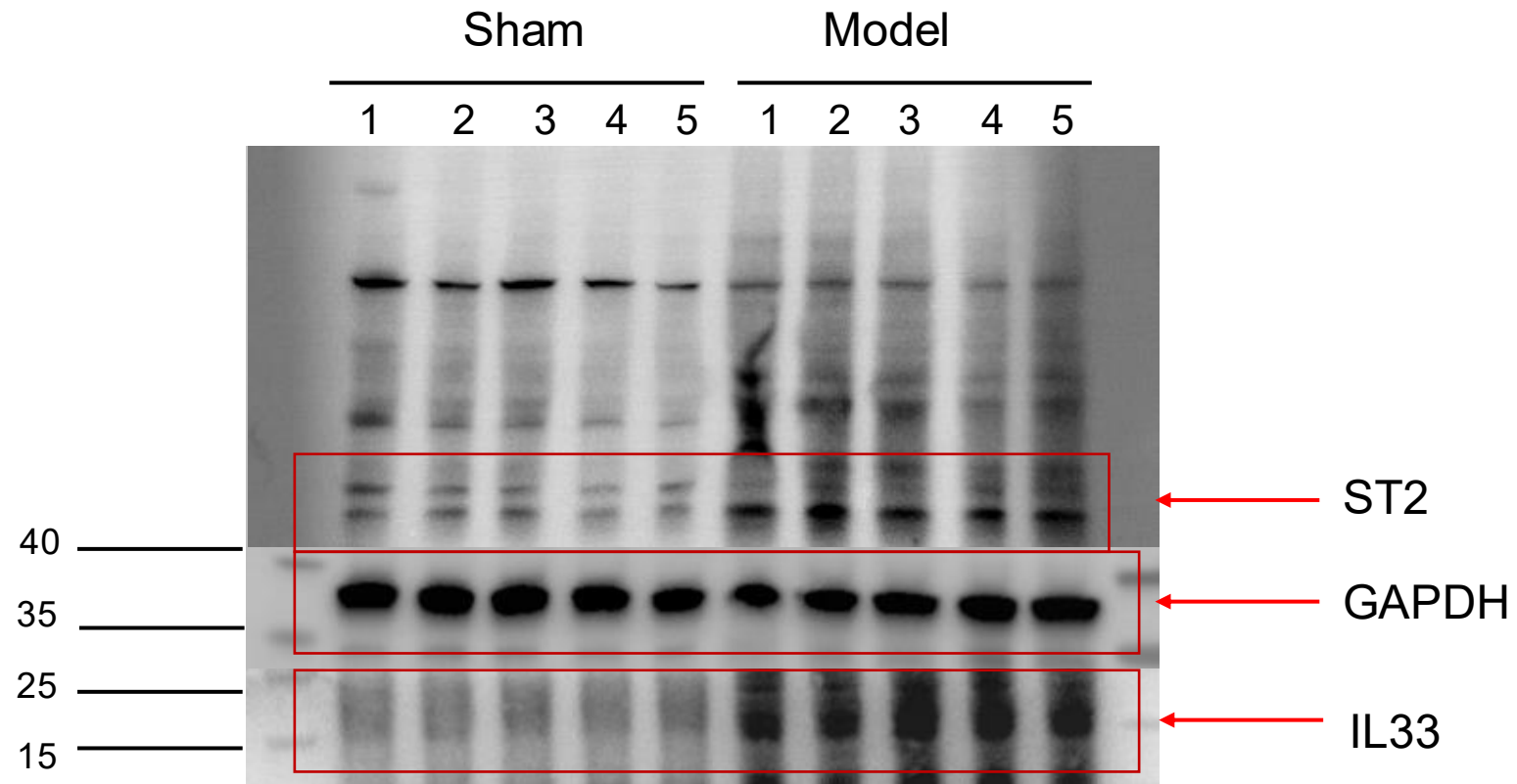

### Figure 5J

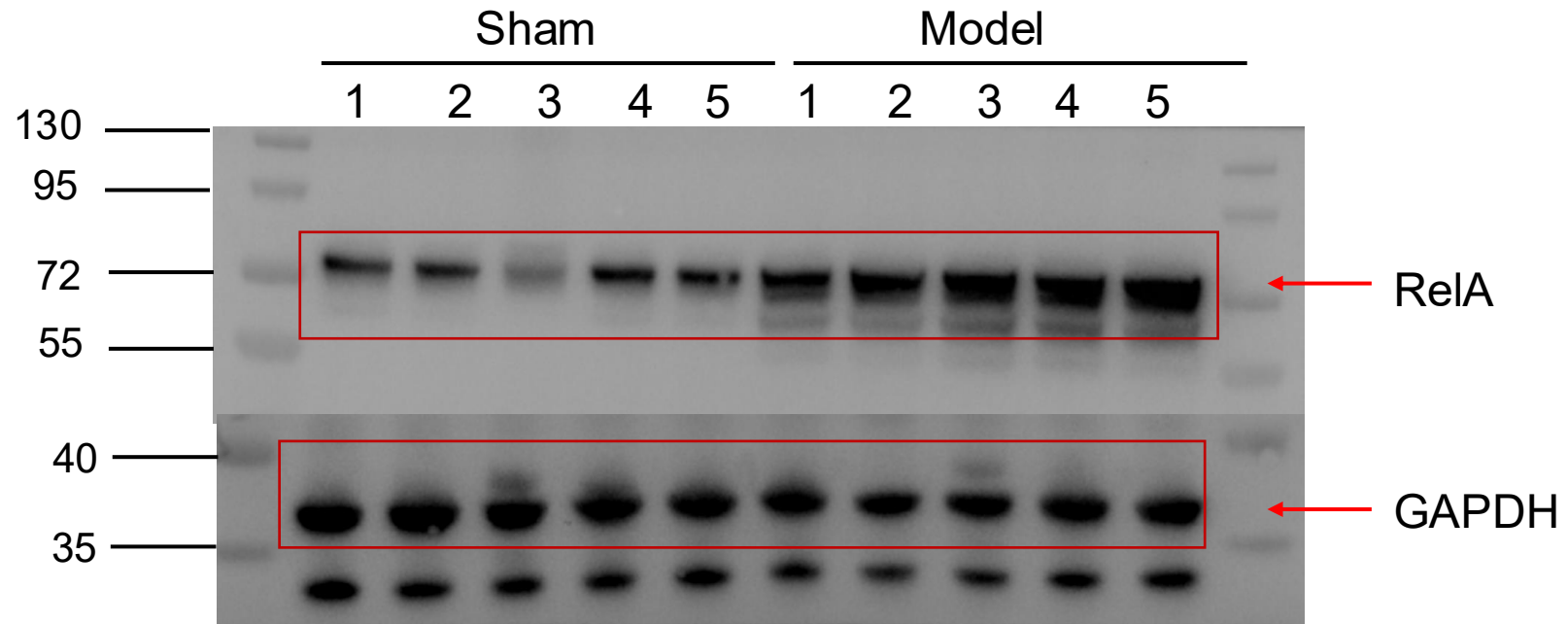

Figure 6K

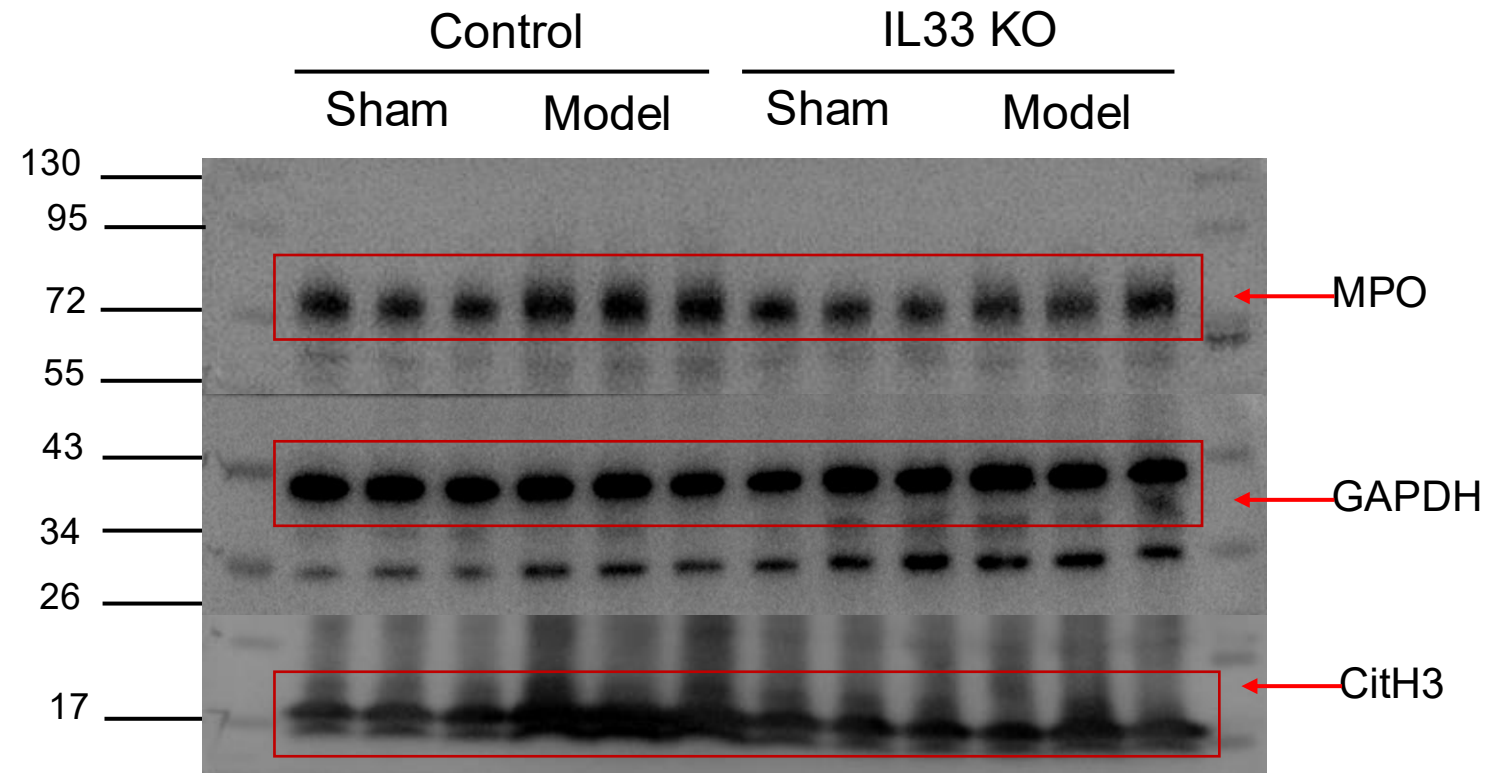

Figure 6N

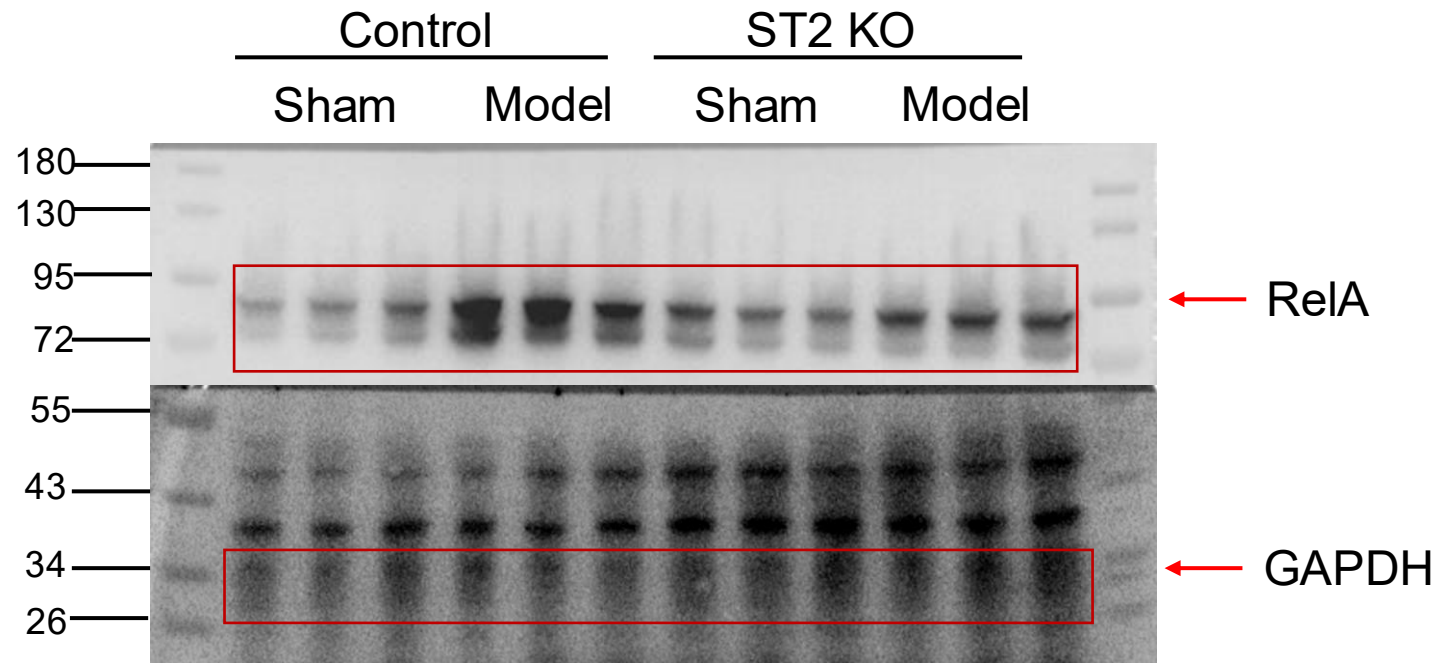

Figure 9L

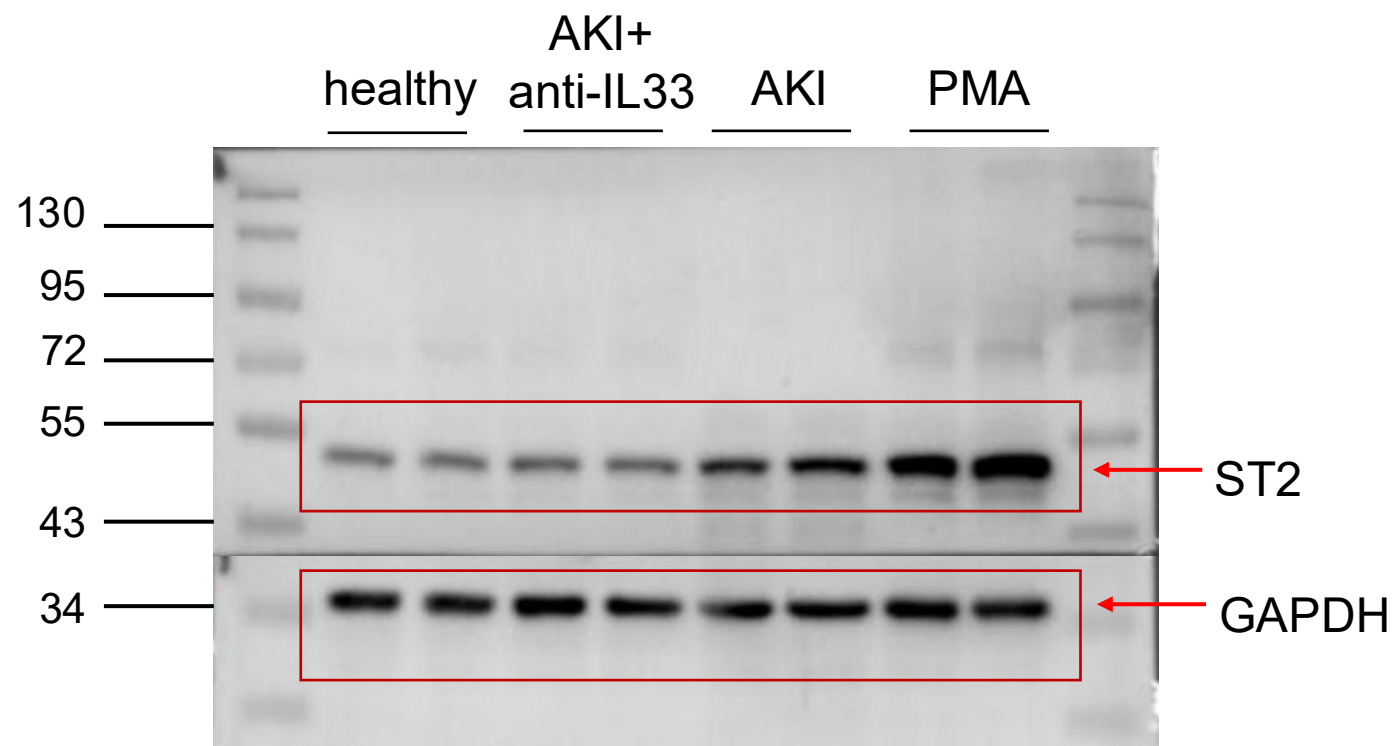

## Supplemental Figure 6M

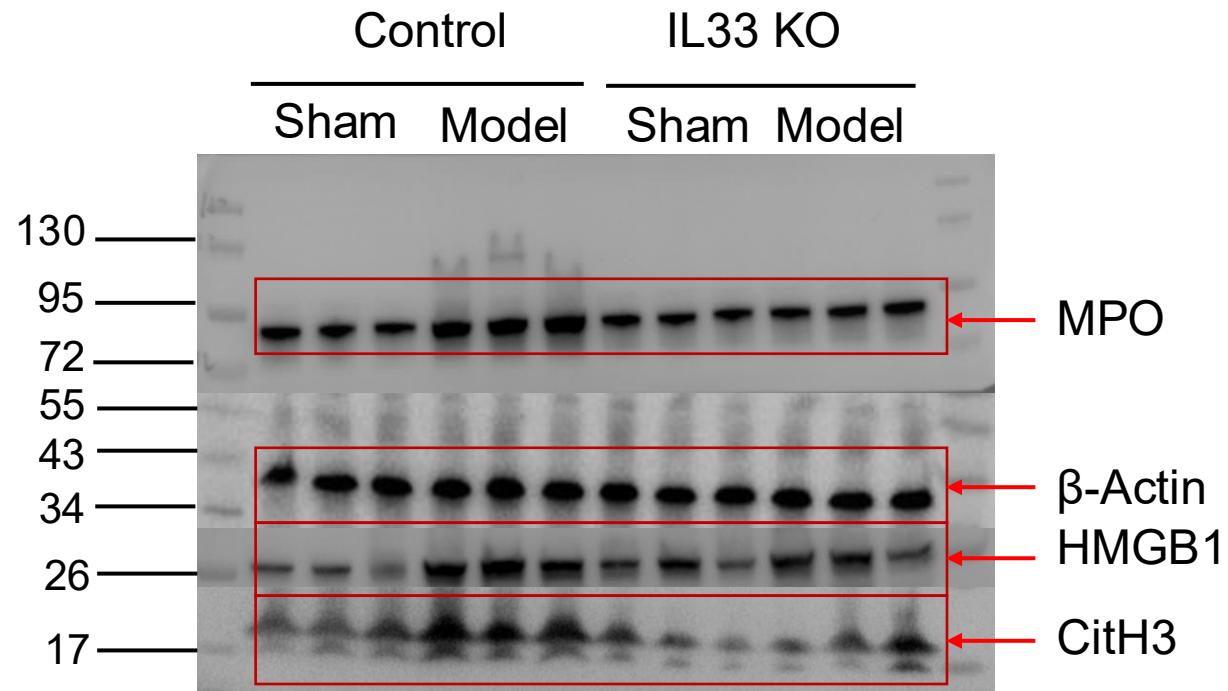

Supplement: Unedited blot and gel images [file jciinsight-11-191979-s287.pdf]
